# Rating scales institutionalise a network of logical errors and conceptual problems in research practices: A rigorous analysis showing ways to tackle psychology’s crises

**DOI:** 10.3389/fpsyg.2022.1009893

**Published:** 2022-12-28

**Authors:** Jana Uher

**Affiliations:** ^1^School of Human Sciences, University of Greenwich, London, United Kingdom; ^2^London School of Economics, London, United Kingdom

**Keywords:** measurement, quantitative, psychometrics, replication, validity, generalizability, construct, rating scale

## Abstract

This article explores in-depth the metatheoretical and methodological foundations on which rating scales—by their very conception, design and application—are built and traces their historical origins. It brings together independent lines of critique from different scholars and disciplines to map out the problem landscape, which centres on the failed distinction between psychology’s study phenomena (e.g., experiences, everyday constructs) and the means of their exploration (e.g., terms, data, scientific constructs)—psychologists’ cardinal error. Rigorous analyses reveal a dense network of 12 complexes of problematic concepts, misconceived assumptions and fallacies that support each other, making it difficult to be identified and recognised by those (unwittingly) relying on them (e.g., various forms of reductionism, logical errors of operationalism, constructification, naïve use of language, quantificationism, statisticism, result-based data generation, misconceived nomotheticism). Through the popularity of rating scales for efficient quantitative data generation, uncritically interpreted as psychological measurement, these problems have become institutionalised in a wide range of research practices and perpetuate psychology’s crises (e.g., replication, confidence, validation, generalizability). The article provides an in-depth understanding that is needed to get to the root of these problems, which preclude not just measurement but also the scientific exploration of psychology’s study phenomena and thus its development as a science. From each of the 12 problem complexes; specific theoretical concepts, methodologies and methods are derived as well as key directions of development. The analyses—based on three central axioms for transdisciplinary research on individuals, (1) complexity, (2) complementarity and (3) anthropogenicity—highlight that psychologists must (further) develop an explicit metatheory and unambiguous terminology as well as concepts and theories that conceive individuals as living beings, open self-organising systems with complementary phenomena and dynamic interrelations across their multi-layered systemic contexts—thus, theories not simply of elemental properties and structures but of processes, relations, dynamicity, subjectivity, emergence, catalysis and transformation. Philosophical and theoretical foundations of approaches suited for exploring these phenomena must be developed together with methods of data generation and methods of data analysis that are appropriately adapted to the peculiarities of psychologists’ study phenomena (e.g., intra-individual variation, momentariness, contextuality). Psychology can profit greatly from its unique position at the intersection of many other disciplines and can learn from their advancements to develop research practices that are suited to tackle its crises holistically.

## Rating ‘scales’: Promises and challenges

Psychology is in crisis, again and anew. Continued debates about replicability (Regenwetter and Robinson, 2017), validity (Newton and Baird, 2016), generalisability (Yarkoni, 2022), robust results (Nosek et al., 2022), preregistration (Szollosi et al., 2020), measurement theories (Trendler, 2019; Uher, 2021c,d) and measurability (Michell, 1997; Trendler, 2009), amongst others, indicate profound problems still unsolved. Astonishingly, however, the widespread use of rating ‘scales’ for quantitative investigations of the complex phenomena of behaviour, psyche and society seems largely unchallenged—even by critics of contemporary practices (e.g., Michell, 2013).

Ratings are popular. Their efficiency to produce large numerical data sets about psychological study phenomena is enormous. Millions of individuals can be studied without any direct contact, nowadays facilitated through online platforms and commercial participant samples featuring desired characteristics (e.g., Amazon’s Mechanical Turk; Anderson et al., 2018). Well-trained scientific staff, expensive equipment, or technical measuring devices are not needed. With ratings, ordinary laypeople can produce numerical data. This spares costly efforts otherwise needed to bring individuals to the lab, elicit the phenomena of interest in experiments, wait for their occurrence in field observations, or deal with the ethical intricacies involved in recording individuals’ everyday activities. Small sample sizes, intense observer training, complicated experimental setups, laborious (e.g., software-based) coding work from multiple observers contrast with the ease of producing large data sets with just some ticks obtainable any time and (almost) any place.

With ratings, it seems, behaviours can be studied even in retrospect (e.g., habitual behaviours in ‘personality’ ratings). Behaviour^1^ researchers, by contrast, must actually see the behaviours and individuals studied—for ratings, this is not needed. Moreover, ratings are used to assess what even the most meticulous recording of physically described and situationally located behavioural acts cannot capture—their appraisal (e.g., normativity, social valence) and interpretation, such as regarding individuals’ intentions, beliefs or feelings that can only be inferred or require self-report. All this information is collected in well-structured data sets, straightforwardly applicable to statistical analysis and seemingly comparable across studies, thus facilitating the generalisation of findings. Compare this with the efforts needed to recruit, meet and interview individuals in one–to–one sessions, to transcribe their verbal data, to code and interpret the textual data thus-produced, and all these efforts to study just small samples with limited options for quantification, comparability and generalisability. No wonder rating ‘scales’ are popular. Indeed, what else could be done with comparable ease and efficiency?

Ease and efficiency—although relevant given limited resources—have never been hallmarks of scientific excellence. Other sciences invested enormous efforts to enable ever more accurate measurement (e.g., 18th century metrologists^2^ measured half the globe to determine the universally agreed length of one metre), to make accessible phenomena previously unexplored (e.g., electron microscope), and to continuously refine their methods to capture even minuscule changes in their objects of research (e.g., spike protein mutations in Sars-Cov2-virus variants). But since the advent of rating methods about a century ago (Thurstone, 1928; Likert, 1932), little has changed in their use to generate data (apart from their digital implementation)—much in contrast to the significant advances made in the statistical analysis and modelling of numerical data thus-produced. Still today, statements or questions (items) describing phenomena of interest are presented to laypersons for graded judgement using fixed answer categories indicating staged degrees (e.g., of frequency) that are commonly considered a ‘scale’ (e.g., ‘rarely’, ‘sometimes’, ‘often’). To enable their application to a broad range of phenomena, contexts and individuals without specifying any particular ones, rating ‘scales’ are commonly broadly worded (Borkenau and Müller, 1991). Colloquial language is used to ensure these ‘scales’ are self-explanatory for laypersons. To further simplify their task, items comprise only short phrases or single words that describe only a particular aspect of complex phenomena, thus presenting chunks of information that can be managed efficiently—mentally by raters and analytically by researchers. The items’ presentation in a predetermined, mixed order is meant to help raters focus on one item at a time without cross-checking their answers for consistency. Indeed, raters are often encouraged to not ponder too long about an item and to indicate the first answer that comes to their mind. Raters need not even formulate their answers themselves but just to tick off the answer categories provided. Raters’ task, so it seems, could not be made any easier.

The apparent simplicity, however, masks intricate challenges imposed on raters. First, raters must interpret items and answer categories to identify relevant phenomena to be judged (e.g., specific behaviours) and the kind of grading enquired (e.g., frequency). Colloquially worded items, however, reflect broad semantic fields of meaning, which are inherently context-dependent. Therefore, raters must construe for each rating a specific meaning and consider specific phenomena to be judged (Wagoner and Valsiner, 2005; Rosenbaum and Valsiner, 2011; Lundmann and Villadsen, 2016; Uher and Visalberghi, 2016). To assess their current intensity or typicality for an individual, raters must recall, consider and weigh relevant occurrences across different occasions, contexts and even individuals (e.g., for ‘personality’ ratings), form an overall judgement and fit it into the answer ‘scale’ provided (Uher, 2018a, 2021d). But occurrences of behaviour are highly complex on all levels of consideration (e.g., individuals, situations, groups, time; Uher, 2015b), not to mention the many interpretive perspectives one can take for explaining behaviours, such as regarding possibly underlying intentions, goals or feelings. Considering all this on demand and out of context in a longer series of brief, isolated and broadly worded descriptions and without much reflection is quite challenging. No wonder respondents often use mental shortcuts, consider just single pieces of information or rely on semantic similarity, common stereotypes or answer tendencies (Shweder, 1977; Wood et al., 2012; Uher et al., 2013b; Arnulf et al., 2014; Uher, 2018a), leading to countless well-described biases (Tourangeau et al., 2000; Podsakoff et al., 2003).

All this questions the accuracy of rating data for psychological ‘measurement’ as well as their utility for quantitative research on the phenomena of behaviour, psyche and society.

### This article

This article analyses in-depth the metatheoretical and methodological foundations on which rating ‘scale’ methods—by their very conception, design and application—are built. *Metatheory* concerns the philosophical and theoretical assumptions that are made about the study phenomena’s nature and the questions that can be asked about them. *Methodology* concerns the philosophy and theory of the approaches (ways) and methods that are suited to explore these phenomena. *Methods,* in turn, are the specific practices, procedures and techniques that are used to perform the therefore necessary operations (Althusser and Balibar, 1970; Sprung and Sprung, 1984; Kothari, 2004). Methodology and method are often conflated (especially in English-language psychology). This reflects many psychologists’ reluctance to elaborate the philosophical and theoretical foundations of their research practices. Rating methods, for example, are well elaborated but their underlying methodology is not.

The first section outlines the philosophical and conceptual frameworks on which the present analyses are based. This prepares the ground to analyse, in the second section, the conceptual foundations of rating ‘scales’, whereby independent lines of critique from different scholars and disciplines are integrated and complemented with novel ones. The analyses reveal a network of 12 complexes of problematic conceptions and erroneous assumptions that support each other and that are codified in common psychological jargon, making it difficult to be identified and recognised by those (unwittingly) relying on them. Specifically, the conceptual problems entail logical gaps that are masked by ambiguous terms, which invite conflations of their disparate meanings. This necessitates a conceptual back-and-forth switching between disparate elements of research as an intuitive attempt to bridge these gaps. This conceptual switching is similar to that experienced with ambiguous images (reversible figures), which cause multi-stable perception and illusions. But unlike these perceptual illusions and concealed by the ambiguous terminology, this conceptual back-and-forth switching goes largely unnoticed—as does its failure to remedy the logical problems.

Through the widespread and uncritical application of rating ‘scales’ as methods enabling psychological ‘measurement’, these problems have become institutionalised in a wide range of research practices, impacting even scientific activities that should be independent of data generation methods (e.g., choice of research questions). Institutionalised problems cannot be remedied with little quick fixes that many may hope for. The aim is therefore to map out the problem landscape to enable an in-depth understanding of the underlying assumptions and concepts that keep the current problematic research practices in place. In-depth understanding is essential to derive meaningful directions for future developments that are needed to tackle psychology’s crises holistically and that are outlined in the final section.

## The present analyses: Conceptual foundations

To critically analyse the philosophical and theoretical foundations of a research system, the most general assumptions on which these analyses are based must be explicated. They form the axiomatic basis from which the specific assumptions and concepts that are used in these analyses are derived and on which the resulting conclusions are based (Collingwood, 1940). Such explications are not commonly made in psychology, which is symptomatic for the discipline’s neglect of its own philosophical and theoretical foundations. Psychology has been operating for too long on the basis of implicit paradigms that are taken for granted and no longer considered explicitly, thereby relying on too many (meanwhile) hidden assumptions that urgently need reappraisal, critical reflection and even radical change and renewal (Danziger, 1979; Gergen, 2001; Fahrenberg, 2015; Smedslund, 2016; Valsiner, 2017b; Toomela, 2018). Explicating the philosophical and theoretical foundations helps identify where differences in conception and understanding may originate from and highlights problems and inconsistencies in the conceptual foundations of rating ‘scales’ but also suitable alternatives.

### Transdisciplinary Philosophy-of Science Paradigm for Research on Individuals (TPS-Paradigm)

**Figure 1:**
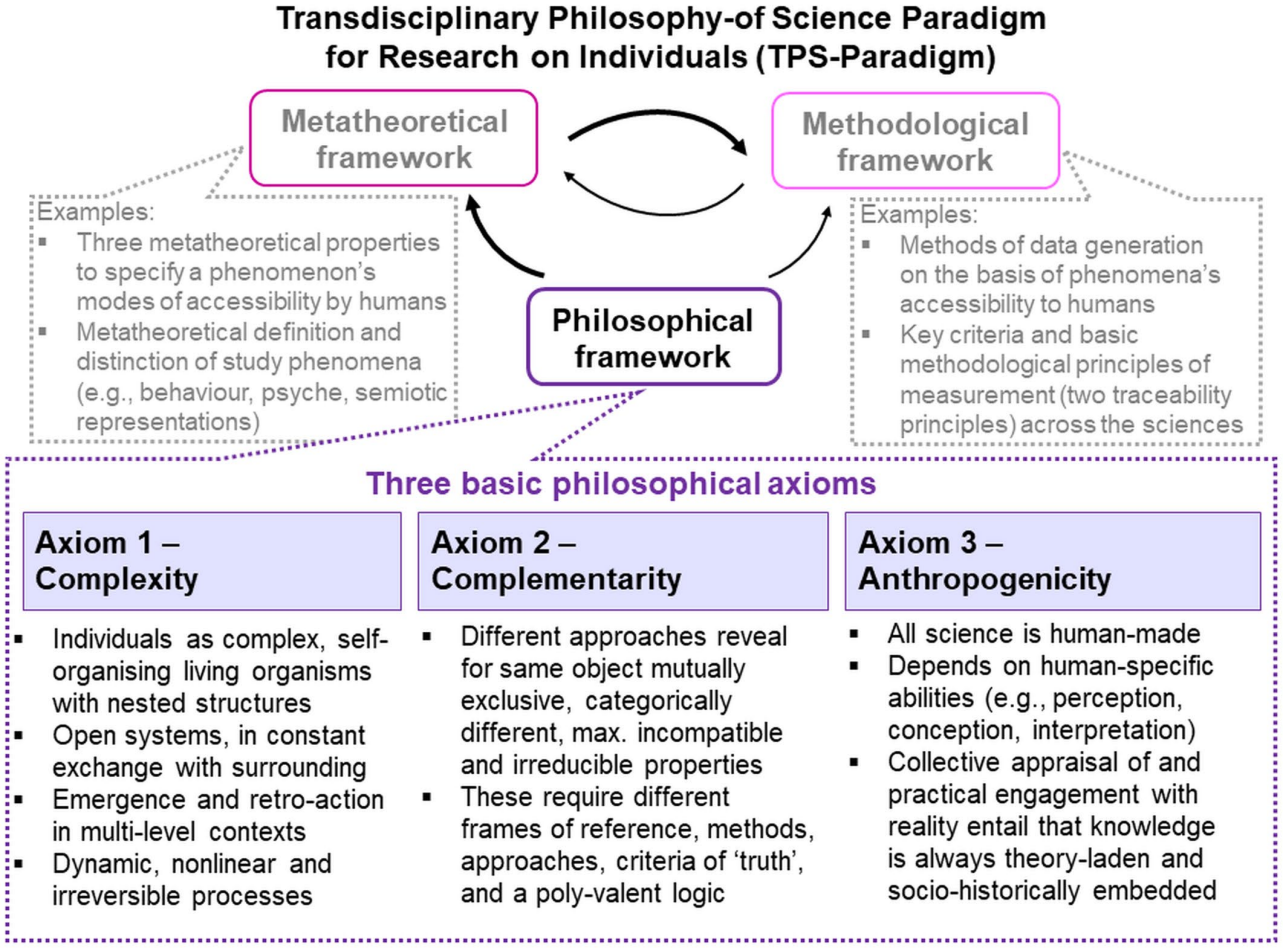
The TPS-Paradigm with its interrelated frameworks and three basic philosophical axioms.

The present analyses are based on the *Transdisciplinary Philosophy-of-Science Paradigm for Research on Individuals* (TPS-Paradigm; for introductory overviews,^3^ see Uher, 2015c, 2018a, 2021b, pp. 219–222). The TPS-Paradigm is targeted at making explicit the most basic assumptions that different disciplines (e.g., psychology, biology, medicine, social sciences, physical sciences, metrology) make about research on individuals involving phenomena from all domains of life (e.g., abiotic, biotic, psychical, socio-cultural). Their holistic investigation, necessitated by their joint emergence in the single individual, poses challenges because different phenomena require different epistemologies, theories, methodologies and methods, which are based on different and even contradictory basic assumptions. To provide conceptual foundations that are suitable for tackling these challenges, established concepts from various disciplines have been systematically integrated on the basis of their underlying rationales and basic assumptions, and complemented by novel ones, thereby creating philosophical, metatheoretical and methodological frameworks that coherently build upon each other and that transcend disciplinary boundaries (Figure 1). These frameworks help scientists to critically reflect on, discuss and refine their theories and practices and to develop new ones, and are therefore well-suited for the present analyses.

The TPS-Paradigm’s philosophical framework with its three basic presuppositions is outlined now. Relevant concepts from the metatheoretical and methodological frameworks are introduced below where needed, including metatheoretical terms and definitions that are used in the TPS-Paradigm to improve clarity and avoid jingle–jangle fallacies.^4^

### Three basic presuppositions

The philosophical framework specifies three central presuppositions that function as the TPS-Paradigm’s most basic axioms for research on individuals—(1) complexity, (2) complementarity and (3) anthropogenicity (Figure 1).

#### Axiom 1 – Complexity: Individuals are complex living systems

As living organisms, individuals are conceived as *open (dissipative) systems* that are in constant exchange with their surroundings but able to maintain themselves through *self-organisation*. Living systems are composed of interrelated elements that are nested on different levels of organisation. On each level, they function as wholes from which new properties emerge that are not predictable from their constituents and that can feed back to the constituents from which they *emerge* (retroaction/transaction), leading to *dynamic, non-linear, dialectical and irreversible processes* of development. With increasing levels of organisation, ever more complex systems emerge that are less rule-bound, highly adaptive and historically unique. Complex psychical systems enable human individuals to be self-reflective, thinking and intentional agents who hold inherently subjective views given their own situatedness in their systemic multi-level contexts (von Bertalanffy, 1937; Shweder, 1977; Capra, 1997; Prigogine and Stengers, 1997; Smedslund, 2004; Morin, 2008; Valsiner, 2021).

#### Axiom 2 – Complementarity: Different approaches can reveal contradictory information about the same object of research

Particular objects of research can be exhaustively understood only by describing two mutually exclusive properties that are categorically different, maximally incompatible with one another, and neither reducible nor transformable into each other, thus requiring different frames of reference, criteria of ‘truth’ and methods of investigation, and that may therefore be regarded as *complementary* to one another. This principle was applied to the wave–particle dilemma in research on the nature of light and has been adapted, amongst others, to the body–mind problem (called psyche–physicality problem in the TPS-Paradigm). In this problem, complementarity takes a metaphysically neutral stance making assumptions of neither ontological dualism nor monism whilst emphasising the necessity for *epistemological* and *methodical* dualism to account for the observation of two categorically different realities that require different frames of reference, approaches and methods. This involves a trivalent or even polyvalent (three- or multi-valued) logic rather than a bivalent (two-valued) logic that many psychologists still (implicitly) apply—a hidden remnant of Cartesian thinking (Bohr, 1937; Fahrenberg, 1979, 2013; Walach and Römer, 2011; Walach, 2013; Uher, 2015c).

#### Axiom 3 – Anthropogenicity: All science is made by humans and thus depends on human-specific abilities

All science is anthropogenic (human-made). Our possibilities to explore and understand the ontological reality in which we have evolved as a species over millions of years are inextricably entwined with and limited by our human-specific perceptual (Wundt, 1907) and conceptual abilities (e.g., interpretations; Peirce, 1958, CP 2.308). Our knowledge about reality is created on the basis of our practical engagement with and collective appraisal of this reality, and is therefore inherently theory-laden, socially embedded and historically contingent (Fleck, 1935; Kuhn, 1962; Valsiner, 2012).

Researchers of individuals face particular challenges because they are individuals themselves and thus not independent from their objects of research. Researchers’ own particular positioning in the world—as human beings, members of particular socio-cultural communities, and individuals—makes them insiders in some regards and outsiders in others. This entails risks for *anthropo-centric, ethno-centric and ego-centric biases* that may (unintentionally) influence their scholarly thinking (James, 1890; Fleck, 1935; Weber, 1949; Danziger, 1997), such as when researchers misattribute properties of their own ingroup to outgroups or overlook outgroup properties uncommon in their ingroup. Such *type-I* and *type-II biases* can influence research on both metatheoretical and methodological levels (e.g., choice of research questions, what constitutes data, analytical approaches or interpretational perspectives taken; Uher, 2013, 2015c, 2020a) and are therefore difficult to recognise.

Anthropogenicity highlights a key challenge for psychologists—the distinction of their study phenomena from their means for exploring these phenomena.

### Psychologists’ cardinal error: Conflating the study phenomena with the means of their exploration—the psych*ical* with the psych*ological*

**Figure 2:**
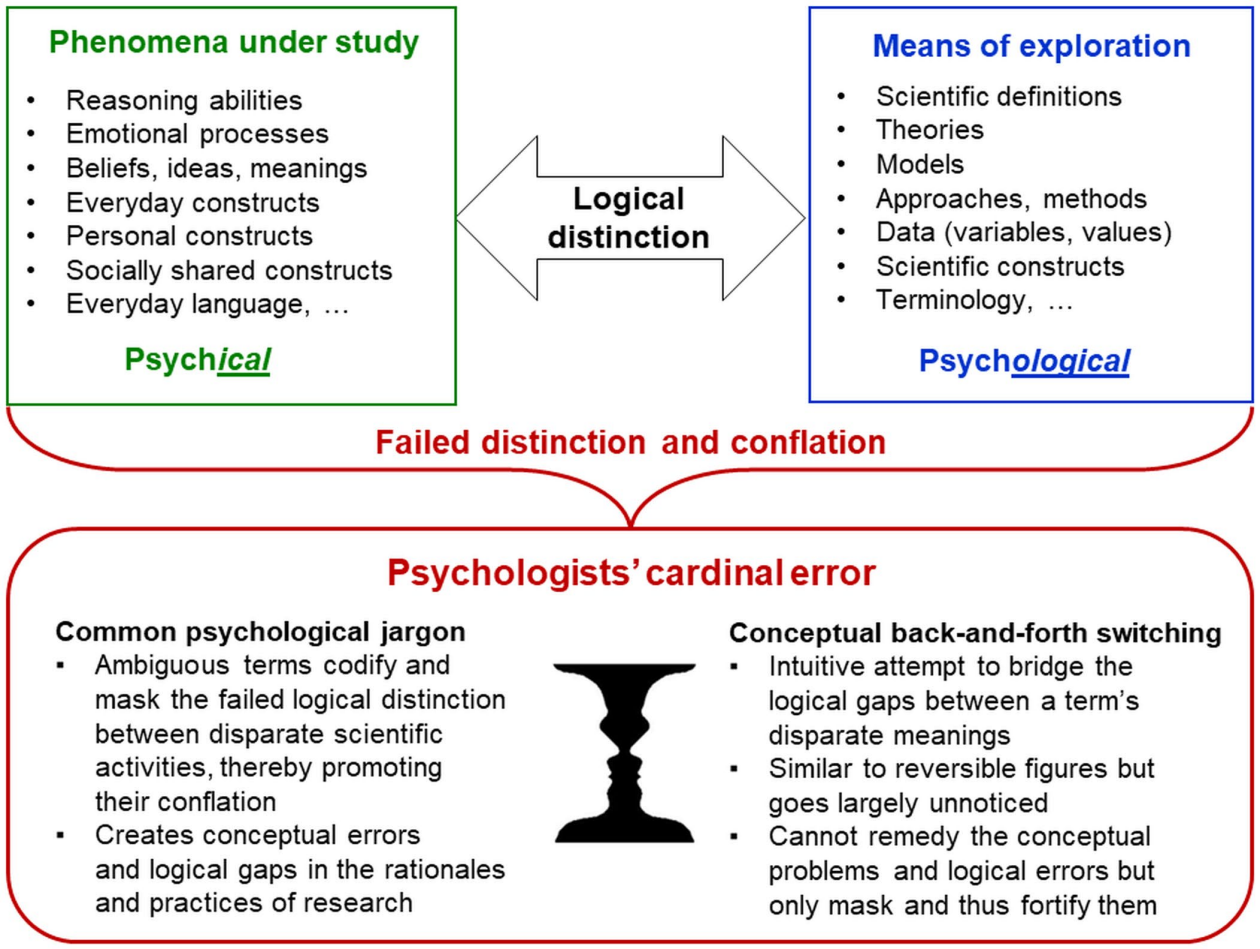
Psychologists’ cardinal error. Abilities of the human mind are essential for any science; empirical research is experience-based by definition. This complicates for psychologists the logical distinction of their study phenomena from their means of exploring these phenomena, which are therefore often conflated—*psychologists’ cardinal error*. Researchers’ intuitive conceptual back-and-forth switching between the different meanings that are being conflated masks the logical gaps created and hinders the recognition of these conceptual errors.

Key scientific activities such as categorising, generalising, conceptualising, abstracting and analysing are abilities of the human mind. Empirical research is experience-based by definition (from Greek *empeiria* for experience). For psychologists—as scientists exploring minds and experience—this complicates the logical distinction of, on the one hand, their study phenomena (e.g., experiences, reasoning abilities, everyday constructs) from their means of exploring these phenomena (e.g., terms, data, methods, scientific constructs) on the other (Axiom 3). In the TPS-Paradigm, this key distinction is emphasised by naming the phenomena of the psyche^5^ in themselves as ‘psych*ical’* (e.g., mental) and the means of their exploration as ‘psych*ological’* (from Greek *-logia* for body of knowledge), as in many non-English languages (Figure 2; Lewin, 1936; Uher, 2016a, 2021b). For example, this article explores psychological problems—professional problems of the scientific discipline—but not psychical problems, which are problems of individuals’ mental health. Naming both^6^ as ‘psychological’ cannot reflect this important difference.

Failure to distinguish the study phenomena from the means of their exploration—here called ***psychologists’ cardinal error***—is reflected in many practices and jargon established in psychology. It entails conceptual conflations of disparate scientific activities, which create logical gaps that researchers’ intuitive conceptual back-and-forth switching between the different activities that are being conflated can only mask but not solve (Figure 2). This logical error has serious implications for entire research programmes because it makes the distinction of disparate elements of research technically impossible, thereby distorting basic conceptions and procedures of science.

## Rating ‘scales’ build on a dense network of 12 conceptual problem complexes

**Figure 3:**
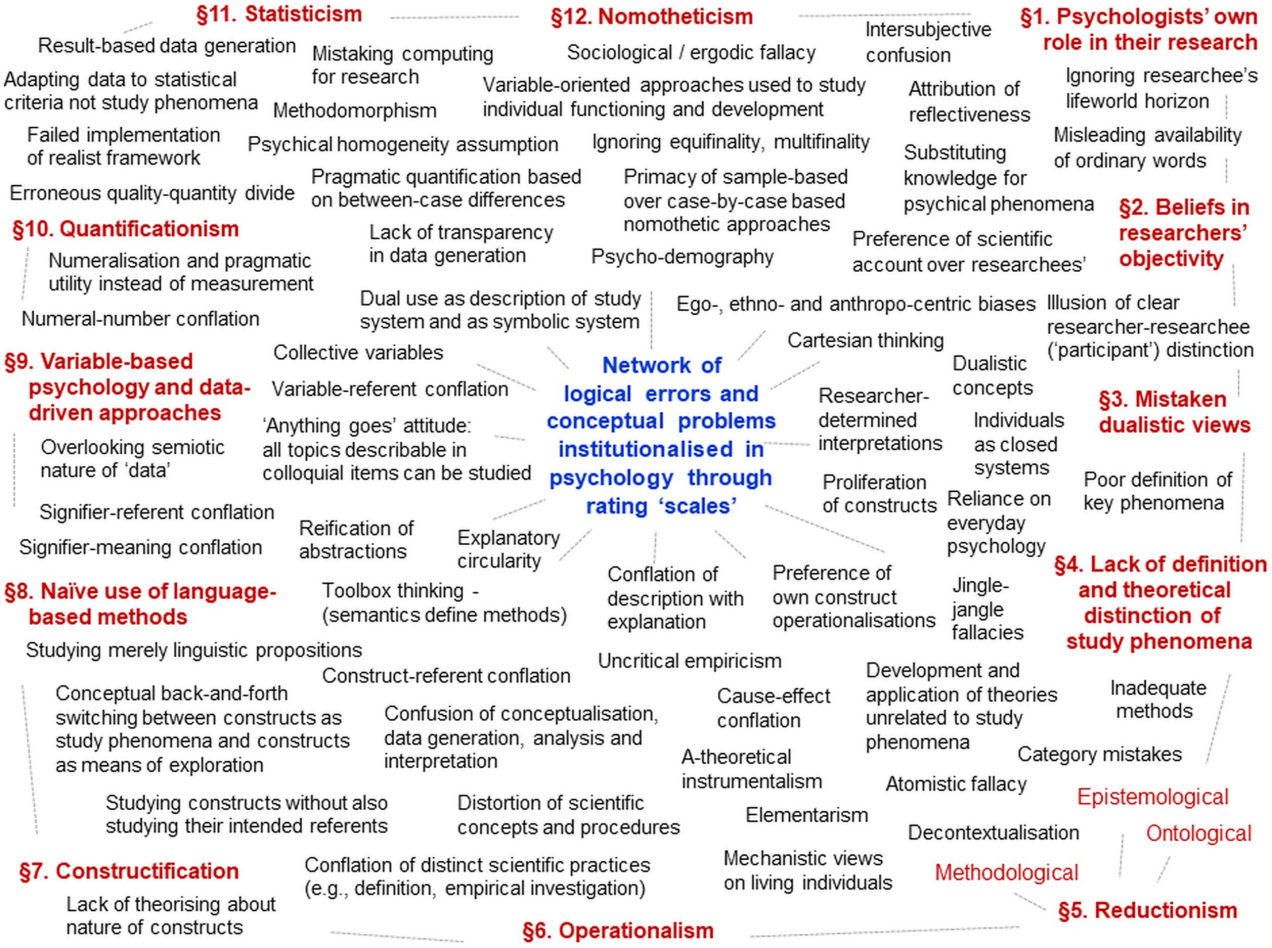
Network of 12 conceptual problem complexes (§1–§12) underlying rating ‘scales’.

Psychologists’ cardinal error is implemented in rating ‘scales’ in numerous ways—12 metatheoretical and methodological problem complexes are analysed in this section. These problem complexes are tightly interwoven, forming a dense network (Figure 3) that underlies current research practices, which therefore appear to be built on a coherent framework for empirical research. But this coherence masks the faulty assumptions, conceptual problems and logical errors on which these practices are based. This makes these problem complexes so difficult to be detected by those (unwittingly) relying on them. They guide researchers’ activities always to the same problematic practices (in different guises), thereby contributing to their perpetuation and psychology’s continued crises.

### Problem complex §1. Psychologists’ own role in their research: Unintended influences

First challenges arise because psychologists are not independent of their objects of research (Axiom 3) whilst, at the same time, essential differences exist between psychologists as researchers and the individuals they research. Specifically, psychical phenomena are accessible (at least partly) only by each individual itself and fundamentally inaccessible by others (Locke, 1999; Uher, 2015a). Therefore, researchers and researchees hold on these phenomena inherently different perspectives, which cannot be shared, enabling observations that the respective other cannot make and may not even be aware of. This disparity can lead researchers of psychical phenomena to mistake their own standpoints for those of the phenomena researched—psychologists’ cardinal error. This entails several fallacies to which psychologists are prone (James, 1890).

Six fallacious assumptions are central (Problem complexes *§1a–f*). Researchers often take for granted that the researchees’ psychical phenomena are similar to their own, thereby attributing onto them their own beliefs about these phenomena rather than investigating these phenomena as they appear to the researchees. This (*§1a*) *intersubjective confusion* may entail an (*§1b*) *attribution of reflectiveness* when researchers assume that phenomena conscious to themselves are also conscious to the researchees, ignoring that psychical phenomena need not include reflective self-awareness whilst they occur. Moreover, researchers study only fragments of their researchees’ psychical phenomena as these are relevant to their research questions, (*§1c*) *ignoring* these phenomena’s relevance within the *researchees’ horizon of their lifeworld*. It is also fallacious to attribute features of psychological theories to the researchees’ psychical phenomena by assuming these are to be understood in terms of categories readily available to researchers, thereby (*§1d*) *substituting knowledge for psychical phenomena*. This also reflects a (*§1e*) *preference of a scientific account over that of the researchees*, which may arise from the researchers’ confidence in their privileged position as experts of psychical phenomena generally but overrides the researchees’ views who hold the exclusive access to the particular phenomena studied. The (*§1f*) *misleading availability of ordinary words* makes researchers prone to suppose a substantive entity existing beyond the phenomenon denoted by a word, ignoring that any psychical phenomenon includes much wider ramifications and connotations than words may suggest but also overlooking phenomena not familiarly recognised in language (see Problem complexes §7 Constructification and §8 Naïve use of language-based methods; Ashworth, 2009; Valsiner, 2017a).

Rating methods involve all these fallacies. Ratings are requested on demand, no matter whether or not raters think of themselves as described, consciously reflect on or actually experience the phenomena described. This reflects an erroneous attribution of reflectiveness (§1b) and researchers’ focus on the described phenomena’s relevance to their own research questions rather than to the researchees’ lifeworld (§1c). Item contents are predetermined by the knowledge underlying the theories, concepts and methods that researchers apply for ‘scale’ development (e.g., for item selection and reduction; McKennell, 1974; Uher, 2015d, 2018a). These practices conflate and (partially) substitute the study phenomena with knowledge that is unrelated to them (§1d). Hence, it is not surprising that item wordings of popular ‘personality’ ‘scales’, even if derived from the person-descriptive words of everyday language^7^ (John et al., 1988), are actually not amongst those used most frequently in everyday life as is often assumed (Roivainen, 2013; Uher, 2013). Rating items are worded as the researchers understand them given their (pre-)scientific knowledge, whereas raters are not allowed to express their views in their own words, reflecting researchers’ preference of a scientific account over that of the researchees (§1e). Item ‘scales’ are presented without much explanation because researchers take it for granted that raters’ understanding of these ‘scales’ is similar to their own. This ignores substantial, context-dependent variations in raters’ item interpretation and use (e.g., Schwarz et al., 1991; Schwarz, 1999; Biesanz and Human, 2010; Rosenbaum and Valsiner, 2011; Lundmann and Villadsen, 2016; Uher and Visalberghi, 2016; Uher, 2018a), reflecting researchers’ intersubjective confusion (§1a) and naïve views on language (§1c), which are further explored below (Problem complex §8 Naïve use of language-based methods).

### Problem complex §2. Beliefs in researchers’ objectivity: Illusions of scholarly distance

These fallacies notwithstanding, and by virtue of their privileged position as investigators knowing (or at least aiming to know) more about the study phenomena than the individuals experiencing them, psychologists typically view themselves as distanced from the individuals and phenomena under study. This disparity, expressed by the term ‘participant’ (‘subject’), creates the illusion of a clear distinction between researcher and researchee, observer and observed. Beliefs in psychologists’ objective, uninvolved stance towards their objects of research are rooted in Cartesian thinking (Westerman, 2014) and natural-science research and became established with the introduction of experiments (Danziger, 1985b).

Wundtian scholars still regarded the participant’s role as source of information more important than the experimenter’s status as operator and attendant and considered both roles as interchangeable (also taking on both roles themselves). This changed fundamentally when Parisian scholars used experiments to study psychopathology (e.g., using hypnosis), which entailed a rigid social differentiation between researchers and the individuals researched. American scholars, in turn, implemented less intense and more impersonal experimenter–participant relations when, commissioned by the American military and government, large-scale investigations shifted psychologists’ focus away from single individuals towards populations of individuals (e.g., through group testing). This established a fixed asymmetry between researchers and researchees; participants became anonymous and distant (Danziger, 1985b). Paper–pencil tests, requiring just minimal instruction, became a favoured medium—and paved the way for rating methods.

Today’s online surveys distance researchers from researchees even further—direct contact is no longer needed, not even administratively. Yet this does not reduce but increase the impact of fallacious assumptions (Problem complex §1 Psychologists’ own role in their research) and of ethno-centric and ego-centric biases (Axiom 3). Problematic findings are therefore not astounding, such as those from international survey panels involving popular ‘personality’ ‘scales’. Instead of showing empirical interrelations as required for psychometric ‘scales’ (see Problem complex §10 Quantificationism), ratings on items used for the *same* ‘personality’ construct (e.g., “is generally trusting” and “tends to find fault with others” for ‘agreeableness’), varied unsystematically, averaging zero across 25 countries (Ludeke and Larsen, 2017). These and further problematic findings (e.g., incongruent factor structures between counties or between different within-country cohorts) challenge these questionnaires’ reliability and validity not only outside of Western, educated, industrialised, rich and democratic (WEIRD) populations but also their adequacy and predictive utility for studying individuals within Western countries (Hanel and Vione, 2016; Laajaj et al., 2019; Condon et al., 2021).

Maximising scholarly distance alienates psychologists from the psychical phenomena under study, which are inherently accessible only to the researchees. Lack of direct contact impedes testing researchers’ (e.g., ethno-centric and ego-centric) assumptions and interpretations, and thus implementation of any corrective means as well as discussions about what objectivity, given the peculiarities of psychical phenomena, could actually mean.

### Problem complex §3. Mistaken dualistic views: Individuals as closed systems

Psychologists’ beliefs in their own objectivity, by virtue of being researchers, entail further problematic conceptions. Specifically, seen from the researchers’ own—supposedly objective—observer standpoint, individuals are often conceived as opposed to, thus separate from the conditions in which they are being studied, as reflected in behaviourist input–output models but also in statistical independent variable–dependent variable (IV–DV) models, amongst others. Individuals are seen as reacting to standardised stimuli that are thought to have the same meaning for all individuals. This conceptual separation underlies, for example, person–situation and person–environment (nature–nurture) research (e.g., in trait psychology) whereby the researchers determine what constitutes a ‘stimulus’, ‘situation’ or ‘environment’, etc. and what meanings these may have for the researchees.

Such dualistic, researcher-determined views reflect a simplistic thinking that facilitates researchers’ work and that enables flexible adaptations to the given knowledge applied (Valsiner, 2017b). But it falls prone to the biases and fallacies on the researchers’ part (Axiom 3; Problem complex §1 Psychologists’ own role in their research). It also overlooks that, as complex open systems (Axiom 1), individuals are interrelated with only those parts of their external surrounding for which they are receptive and with which they can interact given their species-specific, community-specific and individual-specific abilities. Thus, what constitutes an individual’s external context (e.g., a ‘situation’) are just parts of its entire external surrounding.^8^ This external context (from Latin *con* + *texere* for woven together) is defined by characteristics of that individual with which these parts are functionally interrelated (e.g., through its perception or conception of them). Hence, an individual’s external context cannot be conceived independently of that individual although it is—like all parts of the individual’s external surrounding— physically located outside of the individual. The specifics, functions and meaning that an external context has for a given individual may therefore not be apparent for others (e.g., researchers; von Uexküll, 1909; Valsiner, 1997; Uher, 2015a; see also Rotter’s, 1954, ‘psychological situation’ concept).

Psychologists’ common consideration of rating items as (verbal) stimuli to which researchees respond are prime examples of such dualistic concepts. Depending on the researchers’ theoretical views, raters’ responses are used to explore, for example, either individuals’ characteristics (in trait psychology), cultural influences (in cross-cultural psychology), or relative gene versus environment influences (in quantitative genetics). Thus, raters’ responses are flexibly attributed different meanings as needed to match researchers’ theories and to answer their particular questions (see analogously, Bandura, 1996)—reflecting the psychologist’s fallacies (Problem complex §1 Psychologists’ own role in their research). To study individuals’ systemic interdependences with their contexts, it requires *inclusive concepts*, in which relevant parts of an individual’s surrounding—despite their physical independence from the researchee as seen from the researcher’s observer perspective—are identified and conceived only in *dependence* of the researchee’s individual characteristics (see Problem complex §12 Nomotheticism; Uher, 2015a,c; Valsiner, 1997, 2017b).

### Problem complex §4. Lack of definition and theoretical distinction of study phenomena: Conceptual conflations and intersubjective confusions

Psychology’s core constructs (e.g., mind, behaviour, actions) are poorly defined; common definitions are discordant, ambiguous, overlapping and circular (Zagaria et al., 2020). At the same time, terms and constructs for specific psychical and behavioural phenomena proliferate chaotically, creating countless jingle–jangle fallacies (Uher, 2021b). These problems reflect many of the psychologist’s fallacies (Problem complex §1 Psychologists’ own role in their research)—and analogous ones. Specifically, as all socialised persons, psychologists have a complex everyday knowledge, which includes pre-scientific concepts and words for many of their study phenomena (Axiom 3; Uher, 2013). These may be helpful to get research started until more elaborated concepts, terms and definitions are developed. But when researchers substitute their pre-scientific knowledge for their study phenomena (Problem complex §1d) and attribute their own understandings onto their colleagues (“we all know what they mean”; Problem complex §1a), conceptual advancements are hampered.

In the TPS-Paradigm, behavioural and psychical phenomena^9^ are metatheoretically distinguished from one another without implying their ontological separability (Axiom 2). *Behaviours* are defined as the “external changes or activities of living organisms that are functionally mediated by other external phenomena in the present moment” (Uher, 2016b, p. 490). The *psyche* is defined as “the entirety of the phenomena of the immediate [non-mediated^10^] experiential reality both conscious and non-conscious of living organisms” (Uher, 2016a, p. 303). These definitions highlight important points for research on individuals and the present analyses of rating ‘scales’. Behaviours are publicly accessible and physically describable, psychical phenomena are not. Psychical phenomena can be inferred from behaviours but neither are psychical phenomena systematically related to behaviours nor are they contained in the behaviours themselves. Most behaviours are ambiguous because they simultaneously possess various features and can therefore be interpreted differently regarding any possibly associated or causally underlying psychical phenomena (e.g., intentions, goals, feelings; Shweder, 1977; Smedslund, 2004; Toomela, 2008; Uher, 2015c).

A key point behind these metatheoretical definitions is the distinction between *description* versus *explanation*. Behaviours can be described in their momentary physical properties and situational locatedness. But their explanations can go well beyond the here-and-now and can invoke many different *interpretive perspectives*, which all follow logical principles (Kelly, 1955; Smedslund, 2004) yet without being logically determined by a behaviour itself (Shweder, 1977). The same physically described behaviour can be interpreted very differently, depending on the contexts that the interpreting individuals consider for themselves as observers and the individual observed (if applicable). Hence, a behaviour can have different *meanings*, each involving different explanations—which may all be logically justified and thus appear to be reasonable—but of which only some apply in a given case.

The metatheoretical distinction between psychical and behavioural phenomena is also important to explore their relations with one another and with other phenomena internal and external to individuals’ bodies—such as in actions. *Actions* are conceived in the TPS-Paradigm as complex kinds of phenomena comprising (a) behaviours (external changes and activities), their relations with (b) the phenomena of the individual’s external context that are mediating the behaviours’ functionality in the present (see behaviour definition above), and with (c) psychical phenomena directing and controlling these behaviours and their outcomes (e.g., intentionality, goal-directedness). From a certain level of psychical complexity (Axiom 1), individuals are able to conceive of and evaluate the outcomes of their own behaviours and to use these concepts to anticipate possible future outcomes. This enables individuals to adapt their own behaviours, to plan ahead and develop intentions. Anticipated future outcomes can motivate, guide and regulate individuals’ current and future behaviours (Kelly, 1955; Valsiner, 1998; Searle, 2003; Smedslund, 2004; Bandura, 2006; Uher, 2013, 2015a,c, 2018b; Tomasello, 2014). Hence, actions are far more complex and involve more diverse kinds of phenomena than just behaviours.

In common psychological jargon, behavioural and psychical phenomena are often conflated, such as when naming both as (‘inner’ and ‘outer’) ‘behaviours’. This blurs description with interpretation and explanation, opening doors to inferential fallacies and attributional biases (Table 1). It also entails that behavioural and psychical phenomena are methodically treated the same, ignoring their different modes of accessibility, which require different research methods (Axioms 2 and 3; Uher, 2019). This frequent conflation may be an attempt to overcome behaviourist ideas and Cartesian ontological dualism.^11^ It may also derive from everyday experience as everyone can notice the tight links between their own psychical and behavioural phenomena. Everyday language is full of concepts and terms intermingling descriptions with explanations of behaviour. Normal adults often talk as if they had observed others’ psychical phenomena (e.g., intentions)—although causal inferences can be made only on the basis of premises; but these premises often remain implicit and can logically justify alternative inferences as well (Shweder, 1977; Smedslund, 2004).

**Table 1:** Common conflations in psychology.

**‘Behaviour’, ‘Inner behaviour’ – ‘Outer behaviour’**	
Psyche	Accessible only privately; thus phenomena of psychical life, which cannot be directly accessed by others but only by the individual itself; defined as “the entirety of the phenomena of the immediate [non-mediated] experiential reality both conscious and non-conscious of living organisms” (Uher, 2016a, p. 303)
Behaviour	Accessible publicly; thus phenomena that occur external to individuals’ bodies; defined as the “external changes or activities of living organisms that are functionally mediated by other external phenomena in the present moment” (Uher, 2016b, p. 490)
**Cause – effect**	
Cause	Entity providing the generative force that is the origin of something (its effect) and that is thus responsible for bringing about the latter
Effect	Something that is produced by a cause or agent and that is thus a result of these latter; something that follows naturally or logically
**Construct – referent**	
Construct	Conceptual system that refers to a set of independent entities (construct referents) on more abstract levels and that is constructed by filtering relevant information about these referents (e.g., by (de-)emphasising aspects that are considered relevant); thus, it constitutes a conceptual entity, which is not the same as the referents to which it refers and which does not exist in itself as a concrete entity
Referent/Construct referent	Independent entities that are regarded as meaningfully related in some ways or for some purpose although they actually never occur all at once and that are therefore considered only on more abstract conceptual levels as a joint entity (the construct) and that are thus not the same as and different from that conceptual entity
**‘Data’**	
as Study phenomena	The study phenomena in themselves (located, e.g., in individuals) that are to be observed. Not to be confused with psychometricians’ so-called ‘observed’ or ‘manifest’ data, which refer to the raw data and are thus sign systems encoding information about the study phenomena but they are not these phenomena in themselves
as Sign systems	Variables and values (located, e.g., on spreadsheets) carrying information about the study phenomena that are to be analysed. Psychometricians commonly refer to the raw data as ‘observed’ or ‘manifest’ data and the modelled data results as ‘latent’ data; all these data are sign systems but neither the study phenomena in themselves nor structures underlying these phenomena
**Description – explanation**	
Description	Factual statements and discourse intended to give an account of the characteristics of something experienced
Explanation	Statements and discourse that conclusively derive something unknown from known elements such that its origin and development can be recapitulated and made comprehensible
**Individual differences – Individuality/‘personality’**	
Individual differences	Differential patterns describing differences between individuals in a sample (e.g., analysed using variable-oriented approaches); they characterise the population and cannot inform about the single individuals
Individuality/‘personality’	Individual-specific patterns (Uher, 2013) describing an individual’s peculiarities, which implies differences from others over some time but analysed on the individual level (e.g., using person−/individual-oriented approaches) and thus characterising the single individuals
**Methodology – method**	
Methodology	Philosophy and theory of the approaches (ways) and methods suited to explore particular study phenomena
Method	Specific practices, procedures and techniques that are used to perform the operations that are necessary for the investigation, manipulation or elicitation of study phenomena
**Numeral – number** (special case of signifier–referent conflation)	
Numeral	Signifier, sign vehicle, written or spoken entity (e.g., graphemes or phonemes) often used to indicate numbers (quantitative properties) but also letters or just categorical (non-quantitative) information (e.g., phone or house ‘numbers’)
Number	Arithmetical value, mathematical entity describing a quantity
**‘Operational definition’**	
Definition	Formal description of the nature, properties or essential qualities of something
Operationalisation (proceduralism)	Reporting design and method details used for empirical investigation
**Psychical – Psychological**	
Psychical	Phenomena of the psyche in themselves (e.g., mental, emotional, cognitive, experiential etc.)
Psychological	Means used to explore psychical phenomena and the body of knowledge developed about these phenomena (from Greek -logia for body of knowledge)
**Signifier – meaning**	
Signifier	Sign vehicle, written or spoken entity (e.g., graphemes or phonemes) used to represent a referent and its meaning; therefore publicly accessible
Meaning	Sense, purpose, significance, intent or definition that something (e.g., a word, action, or concept) has for somebody
**Signifier – referent**	
Signifier	Sign vehicle, written or spoken entity (e.g., graphemes or phonemes) used to represent a referent and its meaning; therefore publicly accessible
Referent/Sign referent	Designatum, what is being designated and referred to by the signifier of a sign whether concretely perceivable, conceived, imagined or fantasised (e.g., objects, events, concepts)
**‘Variable’, Variable – referent**	
Variables/Data variables	Sign systems encoding information about the study phenomena for the purpose of recording and analysing this information on symbolic levels in lieu of the actual study phenomena (e.g., using statistics). Confusingly, psychometricians commonly refer to the raw data variables as ‘observed’ or ‘manifest’ variables and the modelled results as ‘latent’ variables; but all these variables are sign systems and neither the study phenomena in themselves nor structures underlying them
Variable referents	Study phenomena about which information is being recorded and explored by means of sign systems

Definitions used in the TPS-Paradigm for Research on Individuals to clarify terms and concepts and to avoid jingle–jangle fallacies and conceptual conflations.

The use of everyday language incorporates these fallacies into rating ‘scales’. Items rarely describe observable behaviours only, such as using descriptive action verbs (e.g., talk). Most items are inferential, such as through trait-adjectives (e.g., jealous), trait-nouns (e.g., opportunist), state verbs (e.g., envy) or interpretive action verbs (e.g., help; Semin and Greenslade, 1985; Semin and Fiedler, 1988). That is, items may require raters to judge phenomena that are actually imperceptible to them (e.g., others’ emotions) or no longer perceivable (e.g., past occurrences to judge habitual behaviours as in ‘personality’ ratings). Inferential and complex wordings do not preclude research, as interpretive analyses of textual materials demonstrate (Fahrenberg, 2002, 2003). But in ratings, they obscure which specific phenomena and which specific aspects of them raters actually consider (Problem complexes §1 Psychologists’ own role in their research, §8 Naïve use of language-based methods and §10 Quantificationism).

### Problem complex §5. Reductionism: Category mistakes, atomistic fallacy and decontextualisation

The interpretation of rating-based findings as reflecting “psycho-physical mechanisms” underlying individuals’ behaviour (common, e.g., in trait psychology) reflects further fallacies—those of *reductionism*. Reduction itself is basic to any science. Approaches and methods to reduce study phenomena, their relations, data, etc. are fundamental for scientific model development (e.g., reduction approaches in taxonomic individual differences research; Uher, 2015d, 2018b). By definition, models are reduced (less detailed) representations of complex parts of reality (Axioms 1 and 3). Hence, not all reductions are wrong; but some are fallacious (Fahrenberg, 2013)—here called *reductionism*.

Three forms of reductionism—ontological, epistemological and methodological—are common. *Ontological reductionism* refers to claims about the relations between phenomena whereby it is assumed that complex phenomena can be described in terms of simpler, more fundamental ones. An example is the idea that psychical phenomena would constitute just neuronal firing through electric impulses and neurotransmitters (Brigandt and Love, 2022).

*Epistemological reductionism* is the claim that knowledge about one scientific domain, typically about higher-level phenomena, can be reduced to another body of scientific knowledge, typically about a lower or more fundamental level. An example is to assume that higher-level phenomena could be explained by lower-level phenomena, such as psychical phenomena by underlying neurophysiological phenomena. But Wundt (1902-1903) already highlighted that, even if brain processes would be as clear to us as clockwork, this could not elucidate the interrelations of psychical phenomena in themselves. Analogously, when asking about an object’s weight, the statement “it is red” provides no answer—this constitutes a *category mistake*. Mass and colour are different categories and belong to different systems of description; weight cannot be expressed in terms of colour. The popularity of analogous statements about psychical and physical (e.g., neurophysiological) phenomena does not make them any more true. These phenomena are complementary—both are needed for comprehensive accounts of individuals but one cannot be reduced to or transformed into the other. Such attempts constitute a category mistake. Psychical and neurophysiological processes require different frames of reference, systems of description, epistemological principles and perspectives, which cannot be reduced to each other (Axiom 2; Walach and Römer, 2011; Fahrenberg, 2013; Walach, 2013). For empirical research, scientists may focus on just some kinds of phenomena, thereby blanking out others, such as neurophysiologists and cultural psychologists do. But holistic accounts of individuals always require knowledge of all the different kinds of phenomena occurring in (relation to) them.

*Methodological reductionism* is the claim that complex systems are most fruitfully investigated at the lowest possible level and could thus be understood by dissecting them into their supposedly isolable building blocks. Such *mechanistic* and *elementarist* views may be useful to explore invariant physical phenomena. But studying elements regardless of their interrelations with other elements and of the contexts in which they occur, meets its limits in living systems (Axiom 1). Knowledge about a cell’s *decontextualised* parts and biochemical components does not reveal how they function together in the intact living cell (Rothschuh, 1963; Brigandt and Love, 2022). Elementarist reductionism reflects the *atomistic fallacy* whereby, from information obtained at lower levels, incorrect inferences are made at higher levels of organisation (Diez Roux, 2002). In psychology, elementarist reductionism is reflected, for example, in Western psychologists’ categorisations of psychical phenomena into memory, perception, motivation, emotion, language etc. and their treatment as separate processes—assuming their study in isolation from their contexts could still reveal meaningful information about their functioning in the individual (Danziger, 1990). Elementarism allows researchers to explore only problems of structure, which are analytic and descriptive, but not problems of process and functioning, which are causal (Bartlett, 1932/1995).

Rating items build on the atomistic fallacy. They are seen as manageable chunks of information that could be understood in isolation from the contexts in which they are used—such as the other items, the raters interpreting them, the specific phenomena that raters decide to judge, those they may consider for comparison, the explanatory perspectives they take on them, etc. Decontextualisation could hardly be any more radical. These decontextualised chunks of information are then put together again using statistical procedures, thus using knowledge that is unrelated to the study phenomena described in the items and the contexts of their use (Problem complexes §1d Psychologists’ own role in their research; §3 Mistaken dualistic views and §11 Statisticism). The popular interpretation of statistically reduced rating data as reflecting “psycho-physical mechanisms” that are heritable, universal and evolutionarily adaptive (e.g., ‘traits’; McCrae and Costa, 2008; Buss, 2009) builds on several reductionist fallacies.

Elementarist reductionism is tightly linked to operationalism.

### Problem complex §6. Operationalism: Logical errors and impeded theory development

Wundt developed substantial theoretical and conceptual frameworks for an enormous breadth of psychical phenomena, ranging from psychophysics to cultural psychology (Völkerpsychologie). But many of his concepts were too sophisticated for extensive empirical investigations (Danziger, 1979; Fahrenberg, 2019). Behaviourists, in turn, rigidly avoided altogether to conceptualise phenomena that are inaccessible in others. To establish the fledging discipline’s empirical research on its primary study phenomena, psychologists turned to operationalism from physics (Bridgman, 1927) and adapted it to their purposes in their own specific ways (Feest, 2005). Operationalism seemed to offer a solution, enabling both empirical research and concept development in a surprisingly straightforward manner.

Operationism consists simply in referring any concept for its definition to the concrete operations by which knowledge of the thing in question is had (Stevens, 1935, p. 323).

Its strong links to logical positivism and statistical advancements like factor analysis (Spearman, 1904; Thurstone, 1937) have firmly anchored empiricism in psychology’s methodological conventions. Still today, operationalism is considered an essential feature of rigorous psychological research (AERA, APA, and NCME, 2014).

But operationalism, both as introduced in physics and its psychological variants, has been fundamentally criticised in its most basic assumptions (e.g., Benjamin, 1955; Wallach, 1971; Bickhard, 2001; Feest, 2005). The idea that a study phenomenon’s meaning could be established through the operations needed for its investigation, manipulation or elicitation conflates the study phenomena with the means of their investigation—psychologists’ cardinal error. Specifying operational procedures may help piloting conceptual work about a study phenomenon. But ultimately, operational specifications must be replaced by proper theoretical definitions (Green, 2001; Feest, 2005)—otherwise, this leads to further logical errors. For example, when reasoning ability is operationally ‘defined’ as test performance, this ability cannot also be used to explain this performance. A phenomenon cannot be defined by its effects; such assumptions *conflate cause with effect* (Table 1; Hibberd, 2019).

Moreover, if a construct’s definition depends on a specific procedure, even if just partially, then every change in procedure defines a new concept. This reasoning may have contributed to the *proliferation of constructs* because psychologists tend to disagree much less on their findings than on their construct operationalisations and therefore prefer to use each their own, leading to overlapping constructs and countless jingle–jangle fallacies (Feest, 2005; Uher, 2021b). Yet the idea that every procedural change also defines a new concept contradicts all sciences’ striving to advance their portfolio of methods, including those suitable for studying well-known phenomena (Wallach, 1971). It also contradicts the realist ontology that many proponents of operationalism assume for psychical phenomena (Hibberd, 2019). Psychologists hoped to solve this problem with “convergent operationalism,” which involves multiple independent operations for the same construct (Campbell and Fiske, 1959). But linking constructs with classes of operational procedures does not solve the basic problem that two disparate scientific practices—(1) reporting design and method details, and (2) defining the concept or study phenomenon—are being conflated (Table 1; Green, 2001; Hibberd, 2019).

Rating methods are prime examples of operationalism. As verbal materials, items can be easily reworded and redesigned so that new rating instruments can be created at libitum and low cost—and with them new constructs (Axiom 3). The verbal provision of rating ‘scales’ and brief instructions to raters greatly facilitates the documentation of the operational procedures that are used to specify given constructs (Uher, 2015d). Together with advanced statistical methods and facilitated by their computerised implementation, rating ‘scales’ have therefore become for many psychologists the preferred tool allowing them to conduct empirical research on almost any topic (Lamiell, 2013; Maul, 2017; Arnulf, 2020).

This a-theoretical instrumentalism entails the belief that any rating ‘scale’ that is nominally associated with a study phenomenon could be a valid method for its investigation (e.g., ‘extraversion scale’, ‘neuroticism scale’). This *toolbox thinking* contributes to the proliferation of substantially overlapping constructs and their pertinent, likewise overlapping rating ‘scales’ (e.g., there are dozens of depression and anxiety ‘scales’; Sechrest et al., 1996). Toolbox thinking invites researchers to choose their topics and questions by the methods available rather than vice versa, thereby enacting worlds that are fit for their methods (Law, 2004). But semantics do not define methods. This practice indicates a failure to reflect on and to use language-based methods (Problem complex §8 Naïve use of language-based methods), and contributes to the persistence of problematic research practices and crises in psychology (Toomela, 2009; Valsiner, 2017b).

The common term ‘operational definition’ misleads researchers to assume that mere descriptions of operational procedures could substitute for the theoretical work on a study phenomenon or concept (Table 1), such as when the results of rating operations are used to define or even “identify” constructs—as often done with factor-analysed rating data (e.g., in differential psychology). But scientific definition is logically prior to the scientific task of empirical investigation (Hibberd, 2019). *Operationalisation* in itself, however, is unobjectionable and even needed for construct research (Problem complex §7 Constructification)—as long as it is meant only as specifying the operations or procedures used to investigate (e.g., elicit, test, assess) a research object. In nomological networks, for example, psychologists define the target construct and its sub-constructs in a theoretical framework, specify the operations needed for its investigation in a separate empirical framework and systematically link both frameworks (Wittmann, 1988).

But this *procedurism* is not scientific definition. It is illogic to treat procedurism as constitutive for a phenomenon’s definition or a concept’s meaning (Hibberd, 2019). It also conflates theories about the study phenomena with theories about methods (Uher, 2021c). Theories about study phenomena are tested *via* predictions that can be derived from these theories; this does not require operationalisation. A concept’s theoretical meaning, the testing of hypotheses and theories, and the procedures of measurement or other empirical investigation are not identical. Operationalism conflates these disparate scientific activities, making their distinctions technically impossible and distorting conceptions and procedures of science. “This contributes to the lack of understanding of theory in psychology and to the relative naivety of the theoretical work that exists in psychology” (Bickhard, 2001, p. 42).

Psychologists commonly discuss operationalisation with regard to constructs.

### Problem complex §7. Constructification: Studying constructs without also studying their intended referents

Constructs are central to psychology (Maraun et al., 2009). But research on constructs is plagued by their vague, inconsistent and contradictory definition and use (Lovasz and Slaney, 2013; Slaney, 2017), leaving many psychologists utterly confused:

We do not know what constructs are, that is, we have rarely come across a clear description of what something should be like in order to deserve the label ‘construct’. Constructs, as far as we are concerned, are truly shrouded in mystery, and not in the good old scientific sense that we currently don't know what they are, but will know when we're finished doing the relevant research, but in the sense that we don't really know what we are talking about in the first place (Borsboom et al., 2009, p. 150).

The main source of this confusion is that constructs are sometimes interpreted as theoretical concepts and sometimes as the study phenomena denoted by such concepts and that both interpretations are often conflated (Danziger, 1997; Slaney and Garcia, 2015; Uher, 2021c,d)—psychologists’ cardinal error.

Constructs, like all concepts,^12^ are products of the human mind as are ideas, theories and knowledge (Axiom 3). Thus, concepts are psychical phenomena; this is their ontology—a fact that other disciplines can conveniently ignore and therefore oppose ‘natural’ and ‘real’ phenomena to the concepts designating them. But are psychical phenomena—is the human mind unnatural and not real (surreal)? What difference could there be, ontologically, between scientific constructs and the constructs that people develop in everyday life (see Kelly, 1955)? They may differ in complexity, coherence of linguistic codification and use. But both can only be thought and conceived by persons—and both even by the same person. The difference between them is thus not in kind; a distinction is made only for epistemological purposes. Constructs do not exist outside of the realm of psychical phenomena (Axiom 3)—a first challenge inviting psychologists’ cardinal error (Uher, 2021c,d).

A construct is a *conceptual system* that *refers to* a set of entities—the construct *referents*—that are regarded as meaningfully related in some ways or for some purpose *although they actually never occur all at once* and that are therefore considered only on more abstract levels as a joint entity. That is, constructs do not exist as concrete entities in themselves; they are only thought of as entities—they are *conceptual entities*. For example, the construct ‘intelligence’ may refer to the entirety of a person’s problem-solving abilities, but these abilities can never be observed all at once. The referents of the construct ‘climate’—an area’s long-term weather conditions—cannot be observed all at once either.

The conceptual nature of constructs has three important implications. First, researchers can empirically study only a (manageable) subset of a construct’s referents that they choose to serve as construct *indicators* (e.g., test items), whereas the (hypothetical) universe of a construct’s referents and their essential features form the basis of its theoretical definition. This highlights again the difference between construct definition and operationalisation (Problem complex §6 Operationalism). Second, as conceptual entities, *constructs can refer to entities of all kinds* (e.g., abiotic, biotic, psychical, social, cultural). Specifically, a construct’s referents can involve various entities of the same kind of phenomenon (e.g., various problem-solving abilities in the construct ‘intelligence’) but also entities of heterogeneous kinds of phenomena (e.g., behavioural, psychical, and physiological phenomena in the construct ‘extraversion’). This entails numerous challenges for both conceptual and empirical research.

Conceptually, different kinds of phenomena can be integrated seamlessly into the same construct although they differ in accessibility, thus requiring different methods of investigation (Uher, 2019). Such conceptual conglomerates of heterogeneous referents are *blended constructs* (Uher, 2018b). For example, behaviours are accessible publicly by observation, psychical phenomena only privately through self-observation and self-report, and physiological phenomena require physical measuring devices. A construct’s referents may also have forms of occurrence as diverse as discrete objects (e.g., brain structures), instantaneous events (e.g., heart beats) and continuous processes (e.g., thinking). But this does not hinder their conceptual integration into the same construct. Indeed, constructs are indispensable to study processes because, at any moment, only a part of a process exists. Processual phenomena, such as many behavioural and psychical ones, can therefore be conceived only by generalising and abstracting from their occurrences over time (Whitehead, 1929). For this reason, constructs are essential for psychological research (Uher, 2021c,d).

Qualitatively and quantitatively different referents can be conceptually integrated through abstraction. *Conceptual abstraction* allows humans to filter information about complex entities and to reduce their complexity by emphasising some of their aspects and deemphasising others (Whitehead, 1929), depending on their ascribed (ir)relevance for a particular meaning or purpose (e.g., social valence, prediction). For example, to facilitate the distinction between similar individuals or similar experiences, people often exaggerate in their constructs minor differences (e.g., between individuals or groups) that are considered to be socially relevant and that then appear, in people’s minds, to be much larger than can actually be observed, thereby acquiring *salience* (Lahlou, 1998; Uher, 2015c).

All humans develop constructs (Axiom 3)—individual theories to describe regularities occurring in their daily lives and to discriminate between experiences they have made, and which are therefore idiosyncratic and personal (Kelly, 1955). Individuals use these *personal constructs* to make predictions, gain cognitive control over future events and guide their own actions. They test their constructs’ appropriateness (viability) for these purposes with new experiences, thereby developing their constructs further, integrating and organising them by their level of generality into complex *construct systems* (Axiom 1). Members of the same community, using their socio-culturally shared experiences, can develop an understanding of others’ personal constructs and the actions derived from them, enabling joint understanding and coordinated action. Constructs that proved to be viable to predict and control individuals’ actions in everyday life—thus, to distinguish between individuals in socially relevant ways and to establish normativity—become *socially shared constructs* (Kelly, 1955) and encoded in natural everyday language (e.g., person-descriptive words; Klages, 1926; Tellegen, 1993).

**Figure 4:**
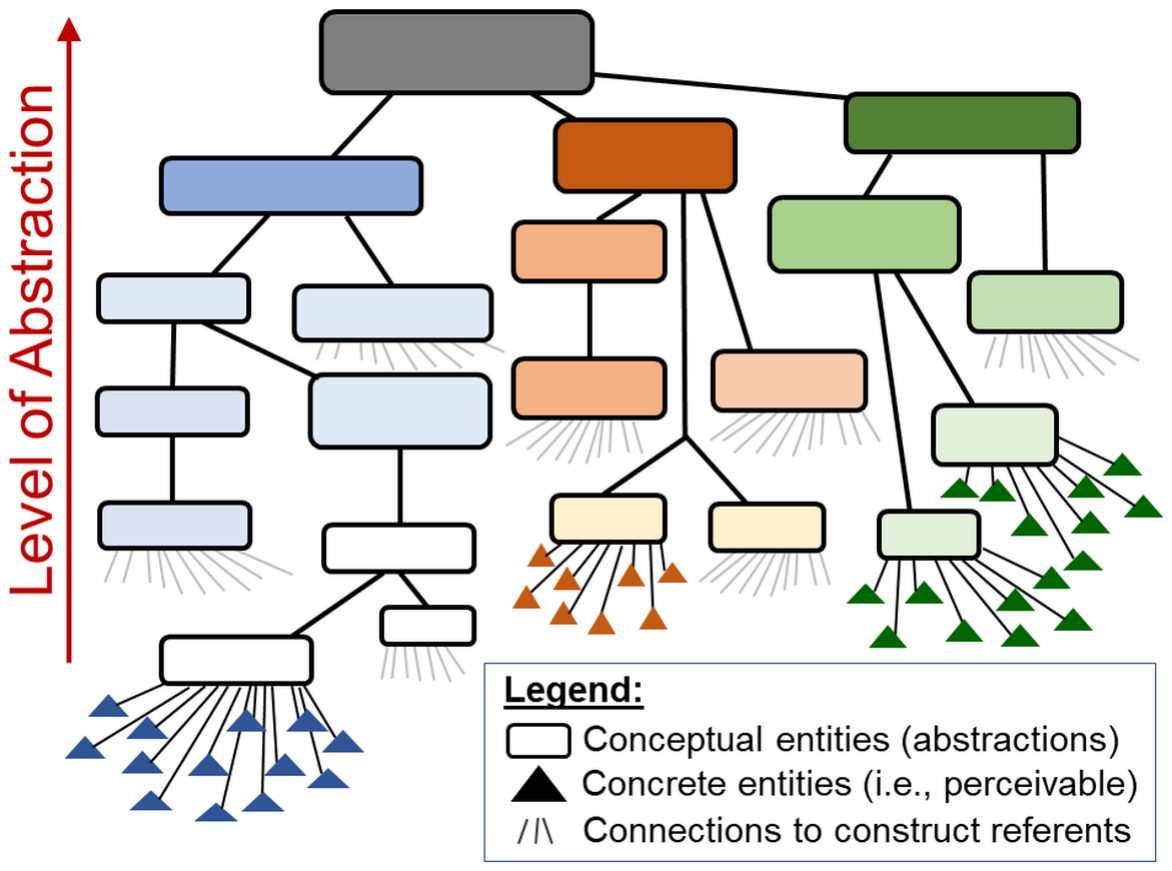
Constructs: Conceptual systems with nested structures. Constructs can be construed on all levels of abstraction. They can refer to concrete entities but also to other constructs, representing their contents and thus the referents to which these constructs refer on higher levels of abstraction. This entails nested conceptual structures (symbolised by words) in which meanings and referents can be ‘inherited’ from the various more specific constructs that they comprise.

Constructs can be construed on all levels of abstraction—from referents that are concretely perceivable at a given moment (e.g., specific behavioural acts) over referents that are conceptual and generalised in themselves (e.g., ‘sociability’) up to referents that are only imagined (e.g., future society) or fantasised (e.g., supernatural beings). That is, constructs can refer also to other constructs representing their content on higher levels of abstraction (e.g., a construct ‘sociability’ may refer to more specific constructs such as ‘gregariousness’, ‘talkativeness’ and ‘approachability’). This entails *nested conceptual structures* (symbolised by words) in which meanings and referents can be ‘inherited’ from the various more specific constructs that they comprise (Figure 4; Uher, 2013, 2021c,d). (For the special role of language therein and their exploration in semantic networks, see Problem complex §8 Naïve use of language-based methods). Constructs and their linguistic labels thus contain complex implicit meanings and conceptual structures (Vygotsky, 1962; Lahlou, 1996). This highlights the third implication of the constructs’ abstract conceptual nature. Constructs *imply more (surplus) meaning* than the concrete indicators by which they can be empirically studied (Problem complex §6 Operationalism) and that therefore cannot be reflected by a construct in the same ways as individuals can perceive them at any given moment (Vygotsky, 1962).

There is no present for all the elements and structures of conceptual systems at once (Althusser and Balibar, 1970, p. 318).

Psychologists’ frequent confusions around constructs can thus be traced back to two interrelated problems, (1) *lack of conceptual understanding of what constructs actually are* and (2) *failure to distinguish scientific constructs from their referents (and indicators)*. Insufficient conceptual understanding involves a lack of awareness that constructs, as psychical phenomena, can be explored in themselves (e.g., people’s everyday constructs in lexical ‘personality’ research; Tellegen, 1993) but that constructs are also important means of exploration (e.g., scientific constructs about everyday constructs, such as the Big Five ‘personality’ constructs). That is, disparate elements of research can be constructs—but, in a given study, the same construct logically cannot be both. Ignorance of this important point led to the implementation of the logical errors of operationalism in psychological research (Problem complex §6 Operationalism). A study’s scientific constructs (e.g., the Big Five ‘personality’ constructs) serve as means of exploration (e.g., as categorical summary statements; Wiggins, 1979) and should thus not be mistaken for the study’s actual study phenomena (e.g., “universals” of human nature that are “invariant across human cultures,” McCrae and Costa, 1997, and thus potentially phylogenetic in origin, McCrae, 2009). Still, scientific constructs can also be explored in themselves—but in other studies using other, higher-order scientific constructs.

These peculiarities make it difficult to distinguish scientific constructs from their referents (and indicators). But constructs only *refer* to particular entities—they *are not* these referents in themselves. Constructs and their linguistic labels (Problem complex §8 Naïve use of language-based methods) facilitate thinking and communication (Vygotsky, 1962). In everyday life, people frequently conflate constructs with their referents (e.g., disease labels taken for illness-causing entities). But *construct–referent conflation* (Table 1) entails serious problems for research because it conflates study means with study phenomena—psychologists’ cardinal error. Recognising such conflations is difficult especially when a construct’s referents are not directly accessible for researchers or conceptual in themselves, as is mostly the case in psychology. Construct–referent conflation was shown to underly confusions about (a) the interrelations between everyday constructs and scientific constructs (Uher, 2013), (b) construct operationalisation, nomological networks and representation theorems (Uher, 2021d), (c) concepts of latent traits, variables and models in psychometrics (Maraun and Halpin, 2008; Uher, 2021c), and (d) disparate notions of the terms ‘hypothetical’ (Lovasz and Slaney, 2013) and ‘unobservable’ (Uher, 2021d) in discussions about constructs.

Rating ‘scales’ promote construct–referent conflation because items are commonly inferential and refer to (often heterogeneous kinds of) study phenomena in more general and abstract terms (Problem complex §4 Lack of definition and theoretical distinction of study phenomena). Such items describe (blended) constructs. Their contents can be judged on the mere conceptual-semantic level (Problem complex §8 Naïve use of language-based methods; Shweder, 1977; Arnulf et al., 2014). But even if raters consider concrete phenomena (e.g., specific behavioural acts), to produce an overall judgement (e.g., on their intensity), raters must implicitly compare them at least over different occurrences, thus over time if not also over different individuals (and phenomena, e.g., other behaviours). That is, ratings inherently involve retrospective considerations and thus abstraction and generalisation. This means, in turn, that ratings cannot capture specific occurrences of phenomena, which, however, is needed for measurement (Problem complex §10 Quantificationism). To study processual phenomena rather than just constructs *about* them, researchers must record the given phenomena as, when and where they occur and over some time using *nunc-ipsum methods* (from Latin *nunc ipsum* for at this very instant; Uher, 2019), such as methods of Ambulatory [ecological momentary] Assessment (Fahrenberg et al., 2007; Mehl, 2017), and must analyse the data thus-obtained for regularities, structures and relations (Van Geert and Van Dijk, 2002; Molenaar, 2008). But in ratings, the phenomena of interest (e.g., social behaviours, emotions) are typically not even present during data generation (e.g., self-rating on screen). The fact that ratings can be generated regardless clearly shows that they are based on raters’ beliefs, ideas and knowledge—and thus reflect personal and socially shared constructs that raters have developed *about* the phenomena described rather than these phenomena in themselves.

Exploring everyday constructs is worthwhile in itself and informative about socio-cultural phenomena. But rating data are often interpreted as reflecting information about individuals’ experiences and behaviours *in themselves*, ignoring that these processual phenomena require methods of data generation other than ratings and therefore remain unexplored (Molenaar, 2008; Rosenbaum and Valsiner, 2011; van Geert, 2011; Uher, 2013, 2016a,b). In consequence, researchers develop scientific constructs without also studying their intended referents—here called *constructification*.^13^ The popularity of rating ‘scales’, thought to enable efficient empirical research on a broad range of behavioural and psychical phenomena, has institutionalised this fallacy widely in psychology. Researchers who rely exclusively on rating ‘scales’ are studying everyday knowledge about their study phenomena, thus laypeople’s generalisations and abstractions with all the biases, inconsistencies and inferential fallacies that these are known to contain (Problem complex §4 Lack of definition and theoretical distinction of study phenomena; Uher, 2013). This is another reason why substantial theories of psychical and behavioural processes (see, e.g., Valsiner, 1997; Sato et al., 2009; van Geert, 2011) are still scarce as some psychologists focussing on constructs and rating-based research noted even themselves (e.g., Haig and Borsboom, 2008; Kyngdon, 2011).

The confusions around constructs are also rooted in the intricacies of language.

### Problem complex §8. Naïve use of language-based methods: Reification of abstractions and studying merely linguistic propositions

Language is human’s greatest invention (Deutscher, 2006). With words, we can refer to objects of consideration even in their absence and although what we say or write (the *signifiers*) typically bears no inherent relations (e.g., resemblance^14^) to the objects referred (the *referents*). This representational function of words arises from socio-cultural conventions that establish signifier-referent relations, which are merely *conceptual* and therefore not directly apparent but which the sign-using person must learn and know (mentally represent).

**Figure 5:**
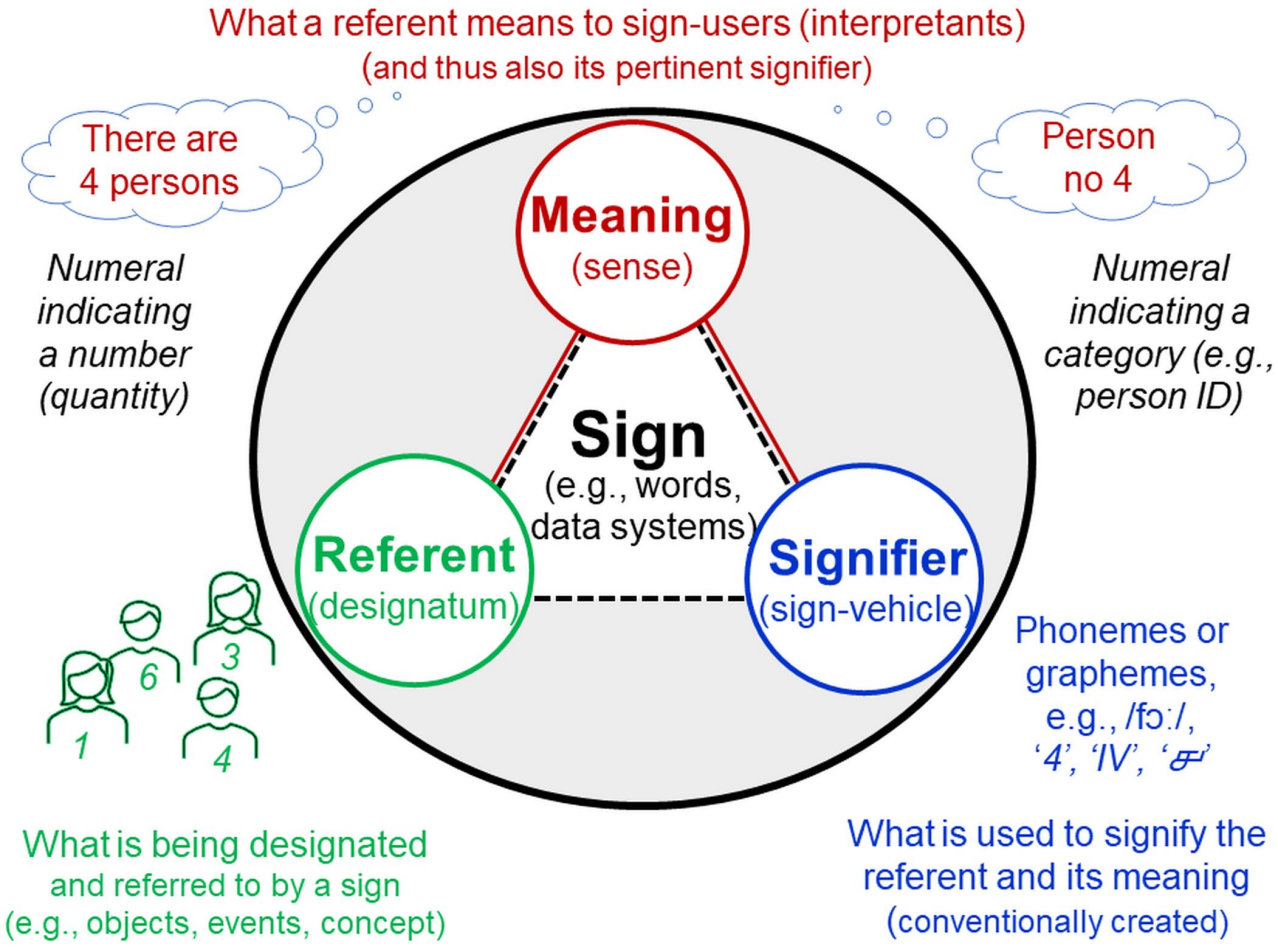
The representational function of language and other sign systems. Sign systems comprise three metatheoretical components: signifiers, referents and meanings. Socio-cultural conventions turn publicly accessible (arbitrary) creations (*signifiers*; e.g., phonemes or graphemes) into sign vehicles that can represent objects of consideration (*referents*) and their sense, significance or purpose for someone (*meaning*) even whilst these objects are absent and without any inherent relations to them (e.g., resemblance).

Each sign (e.g., word, symbol) thus consists of and involves interrelations between three different components: (a) a signifier (sign-vehicle), (b) a referent (designatum) and (c) the meaning (sense) linking them. Specifically, (a) *signifiers* are physical, publicly accessible phenomena that are often arbitrarily and conventionally created (e.g., graphemes, phonemes) and that we use to refer to particular (b) *referents*, which can be anything that persons can perceive and/or conceive of (e.g., objects, events, ideas, concepts), thus any kind of phenomenon.^15^ The (c) *signified* is the meaning that the referents, and thus also the signifiers signifying them, have for the sign-using persons (interpretants)—individually at a given moment but also in socio-linguistic communities over time and contexts—and which is a psychical phenomenon in itself. This psychical component establishes the functional signifier-referent-meaning interrelations^16^ on which signs are based and from which new properties emerge that are not present in each of these three components in itself (Axiom 1; Figure 5). This metatheoretical concept of signs (building on Ogden and Richards, 1923; Morris, 1938; Dewey, 1946; Peirce, 1958; Vygotsky, 1962; Rød, 2004) illustrates that language involves psychical phenomena *in itself* and is thus inseparable from its users’ minds (Axiom 3; Valsiner, 1997). To highlight this, sign systems are called *semiotic representations* in the TPS-Paradigm (Uher, 2015a, 2016b, 2021a).

The representational function of language and other sign systems is essential for abstract thinking because it allows individuals to turn—on mere conceptual levels—perceivable properties (e.g., bitter) into hypothetical objects (e.g., ‘bitterness’), thereby making them conceptually independent of their embodied experience (hypostatic abstraction; Peirce, 1958, CP 4.227). These reified (objectified) properties can become objects of consideration in themselves (e.g., ‘taste’) and can be linked to other perceptions, objects and meanings (e.g., ‘bitter’ as socio-emotional category). This allows individuals to mentally handle abstract ideas and to abstract them further. More abstract words therefore refer to ideas and concepts that are more distant from immediate perception and that cannot be easily traced anymore to their formerly concrete references and contexts. Hence, words carry meanings that are drawn from their logical connections with other words in the semantic space of a language (which can be depicted in *semantic networks*^17^) as well as from the linguistic contexts in which they are being used (e.g., sentence, paragraph). Put differently, words are basic linguistic units carrying meanings that are drawn from the logical, semantic and meaning-making structures of a language. These structures follow particular rules (language-games; Wittgenstein, 1922), which are shared within socio-linguistic communities to enable communication and which are needed to infer the particular meaning that a person may want to express in language (Peirce, 1958; Vygotsky, 1962; Deutscher, 2006; Neuman et al., 2012; Uher, 2015a, 2016b; Khanam et al., 2019).

Rating methods capitalise on these extraordinary abilities of language. Yet many psychologists know surprisingly little about sign systems. Given this, and mislead by the ease of using language (Axiom 3), they often overlook the inherently representational and composite nature of signs—a classic example of competence without comprehension (Dennett, 2012; see Arnulf, 2020). Perhaps therefore, a sign’s most directly apparent component, its signifier (e.g., what is written) is often equated with the entire sign (even if just implicitly). This entails various fallacies, such as when signifiers (e.g., printed item wording) are assumed^18^ to carry *in themselves* the meaning that can be ascribed to them (*signifier–meaning conflation*; Table 1) as reflected in the common idea that standardising rating items could standardise also their meanings for raters (as needed for quantification; Problem complex §10 Quantificationism). Ignoring a sign’s meaning component can also lead to mistake the signifier for its referent, such as when rating items are equated with the behaviours they describe (*signifier–referent conflation*; Table 1) as done in operationalism (Problem complex §6) and leading to constructification (Problem complex §7). But without conceptual signifier–referent–meaning interrelations, a signifier (from Latin *signum* for mark, token)—literally—cannot signify anything (Uher, 2016b, 2018a, 2021c,d). Language-based methods, such as rating ‘scales’, are inherently interpretive and context-sensitive, involving individual and changeable meaning construction (Valsiner et al., 2005; Rosenbaum and Valsiner, 2011). This must be considered when aiming to explore the individual experiences that persons aim to express through language (Stulík, 2022).

Originally, rating ‘scales’ were conceived as capturing just verbal behaviours, whereby verbal declarations were taken as socially accepted symbols for overt acts (Likert, 1932)—an idea refuted almost contemporaneously (LaPiere, 1934). Ultimately, every study phenomenon can be verbally described—otherwise it cannot be researched (Wittgenstein, 1922)—and many psychical phenomena are accessible only through language. Rating ‘scales’ are often treated as if they could capture just any research phenomenon as long as it is describable in small chunks of colloquial language, reflecting an “anything goes” research attitude. Mere hand-movements for ticking boxes (Baumeister et al., 2007) are thereby conflated with raters’ semantically guided meaning construction, beliefs and intuitive judgements encoded in everyday language, which can lead only to pseudo-empirical findings (Smedslund, 1991, 2016). Indeed, in studies using language processing algorithms, more than 86% of the statistical variation obtained in Likert ‘scale’ responses was *a priori* predictable from the items’ semantic fields of meaning (Arnulf et al., 2014).

Availability of a word leads to assume that its referent constitutes a concrete entity (Problem complex §1f Psychologists’ own role in their research). This may be true for words denoting concrete referents that are directly perceivable without reflection but not for “fictitious” words such as those denoting abstractions (Jeremy Bentham, 1748–1832, cited in Ogden, 1932). Linguistic abstractions, such as single word terms for constructs (e.g., ‘openness’), are often mistaken for real concrete entities—the *fallacy of misplaced concreteness* (Whitehead, 1929). This linguistic *reification* promotes the conflation of the study phenomena with the constructs used to explore them (construct–referent conflation; Problem complex §7 Constructification) and may mislead researchers to take descriptions of the study phenomena for their explanation (Table 1), resulting in *explanatory circularity* (Mischel and Shoda, 1994; Uher, 2013). Without considering the complex role and function of language in science and everyday life (Axiom 3), rating-based research runs the risk of studying merely linguistic propositions (Wittgenstein, 1922)—thus, only laypeople’s knowledge *about* the phenomena of interest but not these phenomena in themselves, the fallacy of constructification (Problem complex §7).

### Problem complex §9. Variable-based psychology and data-driven approaches: Overlooking the semiotic nature of ‘data’

Failed distinction of study phenomena from study means is also reflected in the disparate use of the term ‘data’ (Table 1). On the one side, psychologists refer to the study phenomena in themselves (located, e.g., in individuals) as ‘data’ that are to be observed or collected. On the other side, they consider the variables and values carrying information about these phenomena (located, e.g., on spreadsheets) as ‘data’ to be statistically analysed. Whatever meaning one may prefer, ‘data’ cannot refer to both without conceptually conflating disparate elements of research (Uher, 2021c). Analogous problems and conflations occur with the term ‘variable’ (Table 1). The common jargon around variables is intended to achieve a certain formalisation needed for statistical analysis (Problem complex §11 Statisticism). But it implies that either psychologists study only ‘variables’ but not individuals or that ‘variables’ somehow exist in individuals or the world as quantitative entities readily available for statistical analysis (Danziger and Dzinas, 1997; Maraun and Gabriel, 2013; Uher, 2021d).

**Figure 6:**
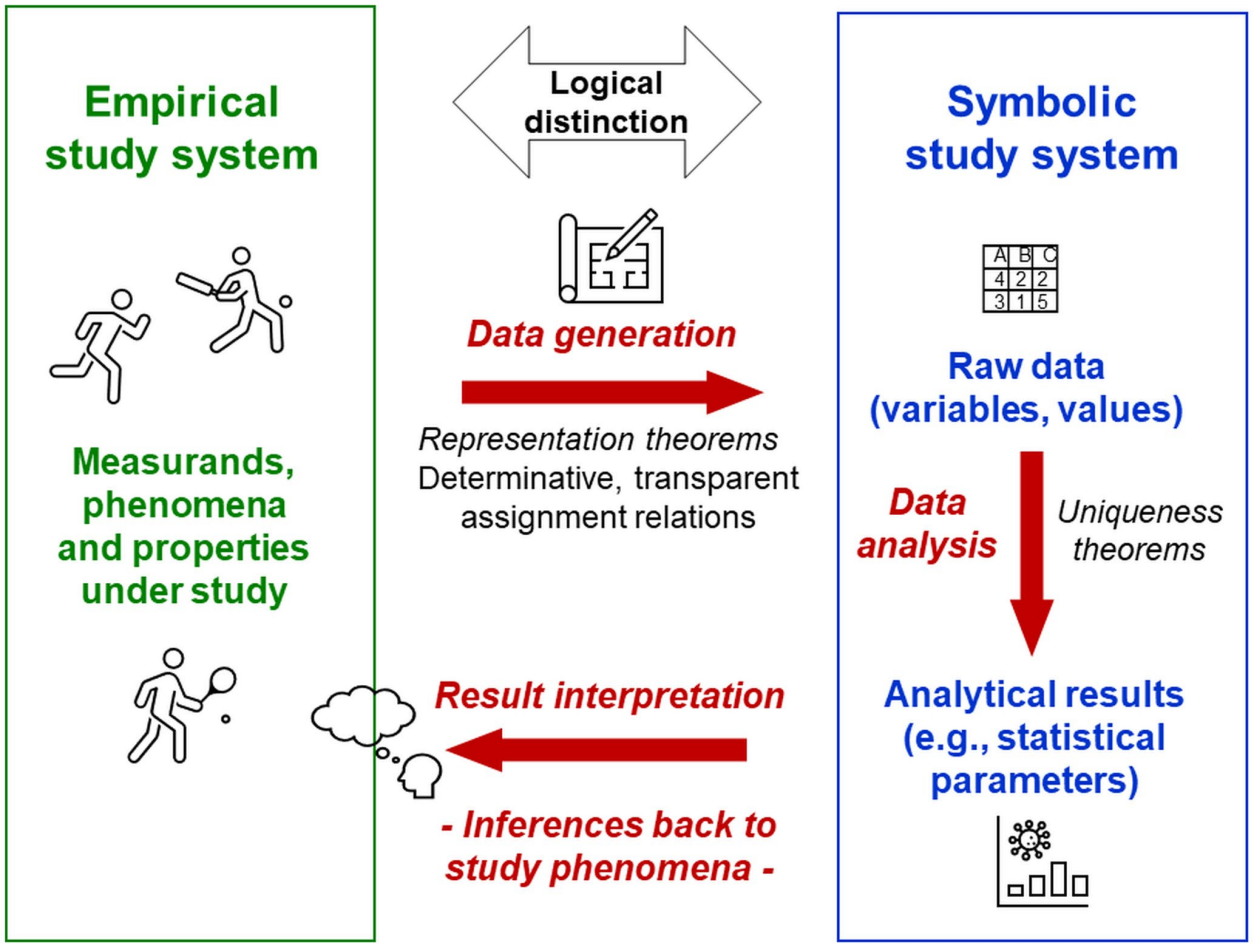
Data generation, data analysis and result interpretation. The principles of *data generation traceability* and *numerical traceability* specify rationales by which transparency can be established in the relations between the empirical study system (e.g., persons’ behaviours) and the symbolic study system (e.g., raw data variables, values). Still needed is the development of analogous principles of *data analysis traceability* specifying rationales for the transformations that are made within the symbolic study system through different kinds of analytical methods. Such general principles will help establish transparency in the analytical results’ relations to the original raw data with regard to the information that these reflect about the measurands and their qualitative and quantitative meanings (e.g., rationales for grouping cases, choosing units of aggregation), thereby guiding researchers’ result interpretation with a clear focus on the empirical study system (Problem complexes §11 Statisticism and §12 Nomotheticism).

*Data* are conceived in the TPS-Paradigm as the sign systems that scientists use to semiotically encode (in *signifiers*) information (*meaning*) about their study phenomena (*referents*; Figure 5). As signs, data can be stored, manipulated, decomposed and recomposed, thus analysed in ways not feasible for these phenomena in themselves (e.g., behaviours). That is, data variables and values are sign systems that are explored *in lieu of* the actual study phenomena and the analytical results obtained from these data, in turn, are used to make *inferences back to* these phenomena (Figure 6). Valid inferences from analytical findings presuppose that it is known what information the data variables and values actually represent, and how exactly they represent the phenomena and properties studied—thus, *transparency in data generation* (§10 Quantificationism; Uher, 2018a, 2021d,c, 2022). This may appear trivial and obvious. But many psychologists’ naïve use of language-based methods (Problem complex §8) and mistaken dualistic concepts (Problem complex §3) lead them to overlook serious problems with their data (Axiom 3).

Data variables that represent information about constructs (e.g., ‘extraversion’; ‘sex/gender’), given their multiple referents, are called *collective variables* (Thelen and Smith, 1994). They encode information such as can be obtained with inferential rating items but also construct indices created by summarising scores across several item variables (e.g., ‘extraversion’ scores). Collective variables entail problems that seriously undermine the meaningfulness of statistical analyses and result interpretations. Specifically, from analysing collective variables, it is impossible to differentiate information about the relations of a single construct referent from those of the conglomerate of all referents considered for a construct. This may mask actual causal relations so that statisticians may misleadingly understand the conglomerate of all construct referents as a cause instead of just one single referent, or vice versa. Causality may also be erroneously attributed to one specific referent although another referent of the same construct is actually important. For example, the blended construct ‘sex/ gender’ may refer to genetic, hormonal, bodily, behavioural and social differences; but which ones are actually relevant and causally related to a study phenomenon cannot be analysed from the corresponding collective variable (for details and further problems, see Toomela, 2008).

Rating ‘scales’ obscure these problems because items are used *both* as description of the study phenomena and as the (item) variables used to encode and analyse information about these phenomena. This dual use, although seemingly efficient, builds on psychologists’ cardinal error, operationalism (Problem complex §6), construct–referent conflation (Problem complex §7 Constructification) and variable thinking (Problem complex §9) and anchors these problem complexes directly into rating ‘scales’. Their dual use leaves the specification of a sign system’s (1) referents, (2) signifiers and (3) the meanings attributed to and thus linking these two latter to raters’ intuitive unknown decisions. This entails serious methodological problems because lack of transparency in *data generation* cannot be remedied by even the most transparent, sophisticated and robust *data analysis* of any preregistered study. Transparent data generation requires specification of (1) the system of the empirical phenomena studied (the referents; e.g., behavioural acts), (2) the symbolic study system used to encode and analyse information about that empirical system (the signifiers; e.g., variable values on spreadsheet), and (3) determinative assignment relations between these two study systems (their meanings), so that the same symbol always encodes the same information about the empirical phenomena (Figure 6; Uher, 2018a, 2021a, 2022).

This idea is basic also to representational theory of measurement, which formalises axiomatic conditions by which empirical relational structures can be mapped to symbolic relational structures (in representation theorems) as well as permissible operations for transforming the latter without breaking their mapping onto the former (in uniqueness theorems; Krantz et al., 1971; Vessonen, 2017). Uniqueness theorems are well-familiar to psychologists (Figure 6), such as for selecting statistical tests that are appropriate for specific data types. But psychologists often overlook (e.g., Borsboom and Mellenbergh, 2004) the fact that explicit representation theorems are basic to any scientific data generation and essential first steps^19^ of measurement (Problem complex §10 Quantificationism; Uher, 2018a, 2020b, 2021c,d). Without appropriate consideration of the inherently semiotic nature of ‘data’ and without explicating their relations to the actual study phenomena, so-called ‘data-driven’ or ‘data-oriented’ approaches can only further institutionalise the problems highlighted here.

### Problem complex §10. Quantificationism: Numeralisation instead of measurement

Much of psychological theory, research and practice relies on quantitative data—thought to be precise, reliable, logic and objective, enabling rigour, standardisation, clear communication and mathematical analysis. The introduction of quantitative approaches was considered an essential means to emulate the physical sciences’ successes and establish psychology as a natural science. Behaviourism and the large-scale assessment industry promoted quantitative approaches as a way of making analyses and decisions independent from the judgement of single experts (Axiom 3). Responsibility for analytical work now lay with instruments, techniques and mathematical-statistical models as unprejudiced tools available for public scrutiny. These scientific methods seemed to enable objective explorations of psychical phenomena in which interpretation and subjectivity hardly played a role anymore (Maslow, 1946; Brower, 1949; Strauch, 1976; Haggerty and Porter, 1997; Westerman, 2006).

These promising prospects drew psychologists’ attention to these tools’ technical correctness, away from questions about their appropriateness and relevance for the study phenomena (Maslow, 1946) and thus also from elaborating the philosophical and theoretical foundations of these tools—that is, their underlying methodology. Quantification became viewed as a positive value *per se* and a quantitative answer as *generally* better than a qualitative one—a belief known as *quantificationism* (Strauch, 1976). Accordingly, psychologists devised quantitative methods that were feasible in their field and that they considered to be analogous to physical measurement—yet without checking if these adaptations actually met (1) the criteria of measurement and (2) the peculiarities of their study phenomena (Strauch, 1976; Valsiner, 2017b; Uher, 2018a, 2020b). Specifically, when operationalists defined a study phenomenon’s meaning primarily by the operational procedures enabling its investigation (Problem complex §6 Operationalism), application of quantitative methods implied the invalid *a priori* answer that “Regardless of what it is, it can be measured—it is a continuous quantity” (Hibberd, 2019, p. 46; Strauch, 1976; Michell, 1997). Operational procedures yielding convergent numerical results were now interpreted as valid instruments for “measuring constructs.” This required (1) the creation of several similar, thus redundant operations for generating quantitative data for the same construct (because, in organisms able to memorise and learn, Axiom 1, possibilities for controlled identical repetitions are limited), (2) methods for analysing the results’ empirical convergence, and (3) rationales for interpreting the found convergences’ meaningfulness.

The manifold nuances of semantics made rating methods ideal to design at libitum^20^ similar and redundant operations to study constructs empirically (Problem complex §6 Operationalism). Aiming to emulate measurement scales, psychologist created verbal ‘scales’ featuring multi-stage answer categories (e.g., ‘rarely, ‘sometimes, ‘often’) that are rigidly scored as numerical values (e.g., ‘1’, ‘2’, ‘3’). Stevens’ (1946) definition of four categories of variables (e.g., nominal, ordinal, interval), indicating information of different levels of complexity (e.g., categorical or sequence information without or with equal intervals), justified the attribution of quantitative properties to such ‘scales’ and the scores obtained with them. To identify sets of item ‘scales’ yielding maximal convergence between their operational results and to analyse the obtained scores’ consistency (reliability), psychologists devised numerous methods of statistical analysis. These mathematical-statistical procedures, although diverse, are largely uncontroversial—much in contrast to the rationales to determine the scores’ meaningfulness (validity) as ‘measurements’ of a construct. Psychologists still debate whether validation involves concurrent or predictive convergence with scores obtained for theoretically related constructs (Cronbach and Meehl, 1955), or rather the scores’ causal relations with the study phenomena (Borsboom, 2005), their plausibility, coherence and appropriateness (Kane, 2013), or the social and ethical consequences of their use (Messick, 1995) and whether validity actually refers to the scores or rather to the instruments used to generate them (Newton, 2012; Kane, 2013).

The basic rationale of quantitative approaches in psychology appears to be coherent at first sight. But it builds on a dense network of misconceptions and conflations that support each other, codified in psychological jargon and woven together through operationalism (Problem complex §6), constructification (Problem complex §7), naïve use of language-based methods (Problem complex §8) and variable thinking (Problem complex §9). This makes it difficult for researchers using these approaches to become aware of the underlying problems and to break out of the intuitive conceptual back-and-forth switching that masks the logical gaps between the different research elements conflated (Axiom 3; Figure 2). These problems therefore emerge time and again in psychology’s controversies and crises—such as in those over psychological ‘measurement’.

#### What is measurement actually? Basic criteria across the sciences

When psychologists use operational indicators to ‘measure’ a construct and interpret the results as reflecting quantifications of ‘it’, they clearly see the construct as their actual study phenomenon—overlooking that scientific constructs are just means of exploration (Problem complex §7 Constructification), thereby committing psychologists’ cardinal error and the logical errors of operationalism (Problem complex §6). The common idea of “measuring constructs” also reflects profound misconceptions about measurement. Psychological ‘measurement’ is often thought to require “the assignment of numerals to objects or events according to some rule” (Stevens, 1946, p. 667)—an idea easily implemented with rating ‘scales’. But this oversimplified definition ignores the basic ideas of measurement, which—across all sciences—are reflected in common interpretations of results that are thought to be obtained through ‘measurement’ (whether or not the specific procedures used actually justify these interpretations). These ideas were therefore used in the TPS-Paradigm to formulate two abstract, general criteria as *basic common denominators that characterise, across sciences, a data generation process as measurement*. These criteria are:

1. *Justified attribution of the results to the measurands* (i.e., the specific entities to be measured) and not of something else (as well)—the ontological claim; and
2. *Public interpretability of the results’ quantitative meaning* with regard to the property measured—the semiotic claim.

These criteria underlie two different yet interrelated methodological principles^21^ for establishing data generation processes that enable measurement and for distinguishing these from other processes of evaluation (e.g., opinion making, assessment). These are the principles of data generation traceability and numerical traceability^22^ (Uher, 2020b, 2022).

#### Data generation traceability: Establishing causal measurand—result connections

The term ‘*measurand’*, although fundamental in measurement terminology (JCGM200:2012, 2012), is unknown to most psychologists. Constructification (Problem complex §7), operationalism (Problem complex §6), inferential rating items (Problem complex §8 Naïve use of language-based methods) and variable thinking (Problem complex §9 Variable-based psychology and data-driven approaches) shifted out of focus concrete study phenomena and with them the necessity to specify the concrete entity to be measured—the *measurand*. But what is it that psychologists actually aim to ‘measure’?

**Figure 7:**
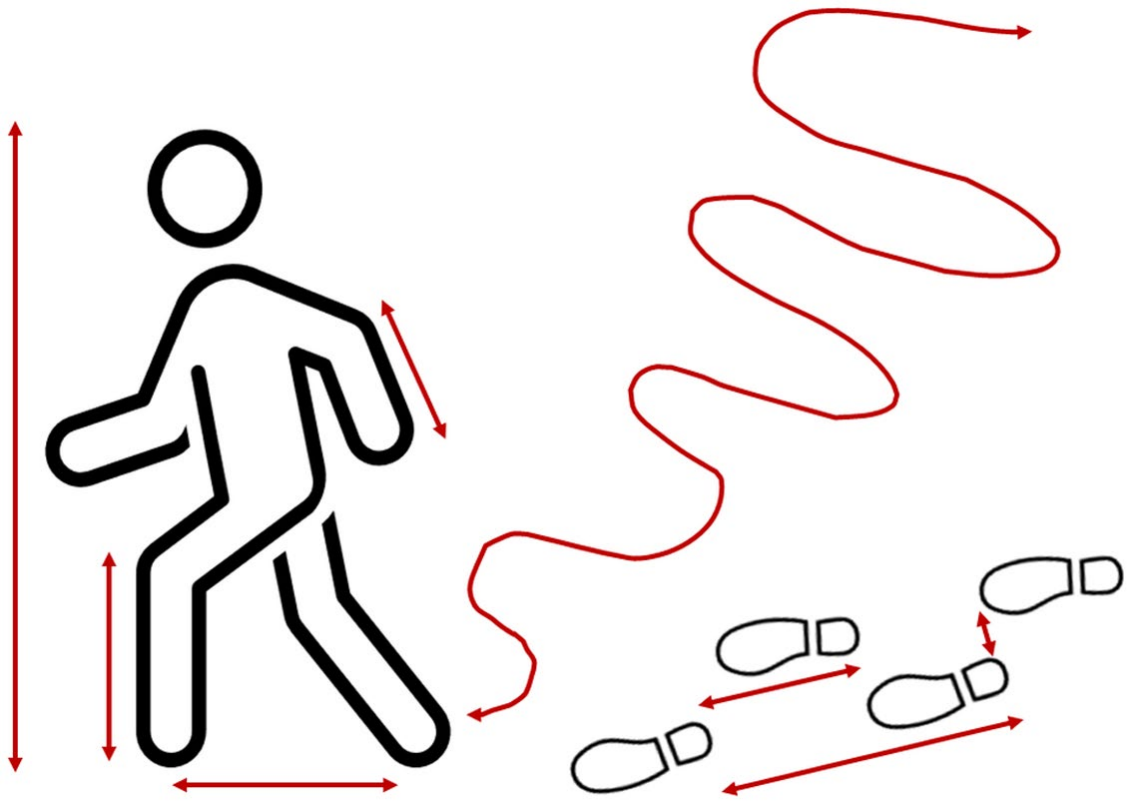
Measurands in individuals. Measurands are the specific entities to be measured; their unknown quantity is to be determined through measurement. Scientists must specify which particular entity of a target property (e.g., length) is the measurand in a study. For example, one cannot “measure an individual’s length” *per se* but only the specific entities of length that it may feature, such as the length of its body height, left lower leg, left forearm, stride, step, distance walked per hour, etc. In rating-based research, by contrast, psychologists often specify just the researchee in general as the entity to be studied (e.g., “measure an individual’s level of ‘activity’”)—a reflection of the inherently conceptual level of consideration taken.

Other than psychological jargon implies (e.g., “measuring behaviour^23^”), objects or phenomena cannot be measured in themselves; only some of their properties can be. Objects and phenomena often feature various properties (e.g., individuals’ bodies feature the properties of length, mass, temperature, etc.; walking behaviour features various temporal and spatial properties, etc.). Therefore, researchers must specify which particular property they study—the *target property*. Of a given target property, in turn, any given study object or phenomenon can feature several entities. One cannot “measure an individual’s length” *per se* but only the length of its body height, left forearm, stride, step, distance walked per hour, etc. Hence, scientists must specify which particular entity of the target property in the given study object or phenomenon is their measurand (Figure 7). Psychologists, by contrast, often specify just the researchee as the entity to be studied (e.g., “measure an individual’s level of ‘activity’”).

**Figure 8:**
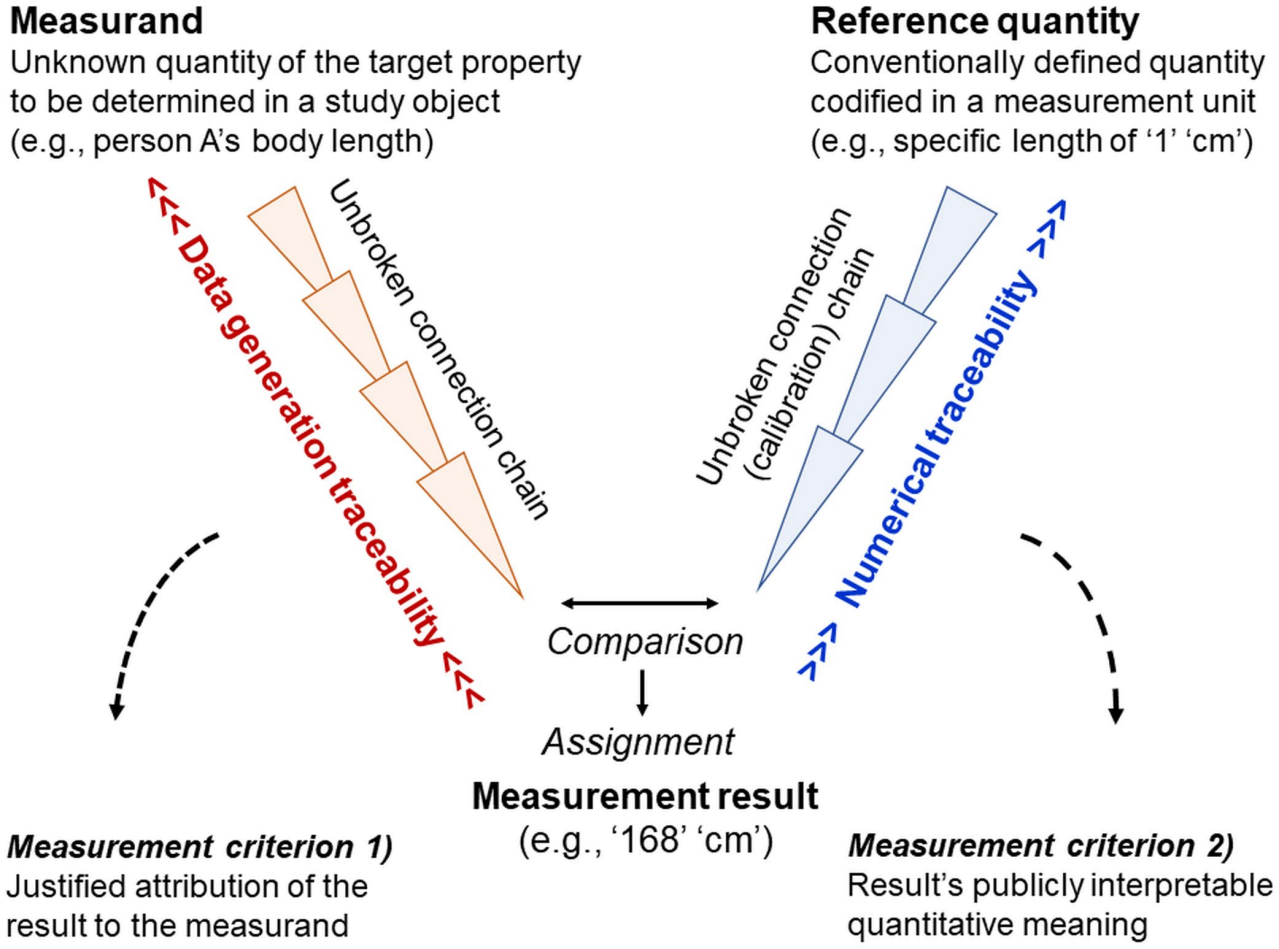
Data generation traceability and numerical traceability. The two methodological principles of data generation traceability and numerical traceability and their relations to the two basic criteria of measurement that are (implicitly) used across sciences.

To justify the attribution of results to the measurands, the entire data generation process must be fully transparent and traceable. This requires operational structures (methods), often implemented through measuring *instruments*,^24^ that enable an empirical interaction with the measurand and that establish *proportional, thus quantitative relations* between the measurand and the result assigned to it. For measurands that are accessible only indirectly, such *causal measurand–result connections* are established through sequential empirical interactions between different properties forming a connection chain, whereby the result of each interaction step depends on that of the preceding step (e.g., indirect measurement). For example, measuring an object’s weight with a spring scale involves the connection chain mass >>> gravity force >>> length of spring deflection (each connected through physical laws) >>> length of extension over the measurement scale (connected through visual comparison) >>> numerical values that measurement-executing persons assign in relation to that scale (connected through semiotic encoding). Unbroken documented connection chains allow a result to be traced, in the inverse direction, back to the measurand, thereby justifying the attribution of this result to that measurand (Figure 8; Uher, 2018a, 2020b).

But what specific values are to be assigned and why?

#### Numerical traceability: Establishing known quantity—result connections

In measurement, numerical values are used to convey in publicly interpretable ways information on the specific quantity determined for a measurand. To establish this semiotic function (see Figure 5), scientists conventionally define for each given target property (e.g., length) (1) particular quantity references (*referents*; e.g., the specific length of 1 metre), which also serve as measurement units (e.g., metre, yard, mile); (2) specific numerical values (e.g., ‘1’, ‘1.09361’) that are used to indicate defined quantities of that target property; as well as (3) empirical interrelations between different quantity references (and between their respective units) codified in non-contradictory mathematical equations (e.g., ‘1’ ‘metre’ = ‘1.09361’ ‘yard’).

To ensure the quantitative *meaning* of these numerical values across time and contexts, primary quantity references (e.g., international prototype metre) are defined and connected with all pertinent working references used for measurement execution (e.g., all yardsticks) through networks of unbroken documented connection (calibration^25^) chains (BIPM, 2006). Implemented in measuring instruments, the reference’s known quantity is set in proportional relation with the measurand’s still unknown quantity, therewith allowing the determination of the latter (Figure 8). Quantity references can be defined arbitrarily^26^ and, albeit in different ways, also in psychology, such as counts of test responses of defined correctness (e.g., in attention or achievement tests; Uher, 2020b, 2022).

These two traceability principles—on their methodological (that is, philosophical and theoretical) level of consideration—guide the necessary methodical (that is, operational and procedural) adaptations to the peculiarities of different disciplines’ study phenomena (considering also further key elements of measurement not discussed here, such as error and uncertainty; see, e.g., Giordani and Mari, 2014). Their implementation in necessarily discipline-specific theories and practices allows measurement results to be traced back to (1) the measurands (data generation traceability) and to (2) reference quantities, which specify the results’ quantitative meaning regarding the target property in publicly interpretable ways (numerical traceability; Figure 8). This allows researchers to make the entire measurement process *and* the results transparent and reproducible (Uher, 2020b, 2021c,d, 2022).

These principles are now used to scrutinise numerical data generation with ratings.

#### Rating ‘scales’: Numeralisation instead of measurement

Inferential items do not specify concrete phenomena and properties to be judged (Problem complex §8 Naïve use of language-based methods), but some answer ‘scales’ indicate a target property, such as frequency ‘scales’ or the popular agreement (Likert) ‘scales’. But can agreement reasonably be assumed to be a property of phenomena as diverse as those described in constructs of ‘anxiety’, ‘honesty’ or ‘extraversion’? In judgements of physical properties, such as of the length of lines in Asch’s (1955) classic experiment on social conformity, it is obvious that agreement is not a property of these lines but of the persons judging them (Axiom 3). But disentangling the mental abilities that are inherently involved in such judgement processes (e.g., working memory, mental complexity, time perception, self-knowledge) from the specific psychical phenomena to be judged (e.g., beliefs, feelings)—thus, the study means from the study phenomena—is difficult. Indeed, to what extend well-documented individual differences in these mental abilities (John and Robins, 2022) influence raters’ abilities to provide graded judgement has hardly been studied (Toomela, 2003). No wonder that, in rating-based research, measurands are commonly left unspecified. Often, the entity to be judged is specified only as the researchee *in general* (e.g., how someone *is*). This inevitably requires abstractions from the momentary occurrences of (psychical or behavioural) phenomena; thus, their consideration at construct level (Problem complex §10 Quantificationism; Whitehead, 1929; Kelly, 1955).

Raters must form and indicate their overall judgements using a bounded set of (mostly) verbal answer categories indicating staged degrees of the enquired property (e.g., frequency) in general, abstract words (e.g., ‘seldom’, ‘sometimes’, ‘often’). But *how often* is ‘often’ for a behaviour to occur given that occurrence rates generally vary between behaviours and situations (e.g., chatting vs. laughing in a café vs. hospital; Uher, 2015b)? Regardless of the *different* phenomena that raters consider for an item and that researchers enquire in *different* items, raters must always fit their judgements into the *same* set of answer categories. That is, raters must assign a broad range of quantitative information *flexibly* to a fixed narrow range of values (e.g., five)—thus, *adapt their judgements to the ‘scale’ rather than to the phenomena and properties to be judged* (Problem complex §1 Psychologists’ own role in their research). This is possible only by constructing for the *same* ‘scale’ category *different* quantitative meanings, which can distort and even inverse quantitative relations (e.g., chatting ‘sometimes’ may actually refer to higher frequencies than laughing ‘often’; Uher et al., 2013b; Uher, 2015b). This fundamentally contradicts the idea of measurement. For accurate and reliable determination of quantities, physical measurement scale units have unchangeable quantitative meanings and pertinent values can be assigned to measurands without upper limits^27^ (Uher, 2022).

Regardless of raters’ flexible assignments, psychologists score the verbal ‘scales’ by rigidly recoding^28^ the same answer category (e.g., ‘agree’) always into the same numeral (e.g., ‘4’). *Numerals* are *signifiers* (Problem complex §8 Naïve use of language-based methods), that is, sign-vehicles (e.g., graphemes, such as ‘4’, ‘IV’, ‘௪’^29^) that can signify *numbers*—that is, arithmetic values indicating quantities—but also just categorical information (e.g., phone or ID “numbers”^30^) or even letters (e.g., Roman numerals; Figure 5). Psychologists actually do not assign numerical values *in relation to* a scale’s units as in measurement. Instead, they *replace the answer units in themselves with numerals*, thereby creating rating ‘scores’ devoid of information regarding both the specific property studied (e.g., ‘4’ *of what*—agreement, frequency, duration or intensity?) and the specific quantity of that property that these numerals are meant to indicate (e.g., *how much* of that is ‘4’?).

A numerical score has no quantitative meaning in itself; a measurement value does—it derives from the measurement unit *in relation to which* the value is assigned (e.g., ‘4’ ‘metre’ ≠ ‘4’ ‘yards’ ≠ ‘4’ ‘gram’) and through which it is conventionally linked with a specific defined quantity of the given target property (numerical traceability). Hence, in measurement, scientists assign not numbers as many psychologists believe (misreading^31^ even Steven’s simplistic definition of ‘measurement’) but numerals, which are defined as quantity values of a particular property (Mari et al., 2015; Uher, 2021c,d). Consequently, there is just one correct numerical value to conventionally indicate a specific quantity—otherwise accuracy and precision^32^ could not be achieved. Rating ‘scales’, by contrast, enable only *numeralisation*—the creation of numerical scores without specified referents—neither any measurands, nor defined reference quantities nor even the property under study (Uher, 2022). Instead, which specific numerals are assigned to verbal answer ‘scales’ depends solely on researchers’ study-specific decisions about the structural data format (e.g., number of answer categories, unipolar or bipolar scoring; Schwarz et al., 1991; Simms et al., 2019). But these decisions have nothing to do with the quantities to be determined. Indeed, psychologists rigidly recode ‘scale’ categories *in the same ways for all items* of a questionnaire regardless of the phenomena described—another instance where they substitute the study phenomena with knowledge unrelated to these phenomena (Problem complex §1d Psychologists’ own role in their research).

Many psychologists seem to be unaware of these problems, believing numerals always indicate numbers (*numeral*–*number conflation*; Table 1; see similarly *numerology* ^33^)—and thus, quantities. But what is quantity actually? How many psychologists can clearly define it? Numeralisation misleads many to believe that quantities could exist and thus be treated independently of their qualities, as reflected in the common yet erroneous polarisation of ‘qualitative’ versus ‘quantitative’ data, methods, etc. This may also explain why psychologists commonly interpret findings from agreement ‘scales’ not as reflecting raters’ levels of agreement, as encoded during data generation, but instead as quantifications of the diverse phenomena *in themselves* that are described in the items (Uher, 2022). This corresponds to re-interpreting a measurement of, let us say, temperature *ad libitum* as one of length, mass or time. Any quantity is always *of something* (Kaplan, 1964). Study phenomena and properties can be identified as such only by their particular qualities; therefore, all quantitative research ultimately has a qualitative grounding (Campbell, 1974). Quantities (from Latin *quantus* for how much, how many) are divisible properties of entities of the same kind—thus, of the same quality^34^ (Latin *qualis* for of what sort; Hartmann, 1964). Entities of equal (homogeneous) quality can be compared with one another in their divisible properties (quantities) in terms of their order, distance, ratio and further relations as specified in the axioms of quantity (e.g., equality, ordering, additivity; Hölder, 1901 ^35^; Barrett, 2003).

What divisible properties (that is, quantities) could agreement ‘scales’ reflect? ‘Strongly agree’ (‘5’) may certainly indicate more agreement than ‘agree’ (‘4’). But could ‘agree’ (‘4’) reflect more agreement than ‘neither agree nor disagree’ (‘3’), often chosen to indicate ‘inapplicable’ (Uher, 2018a)? Does ‘agree’ (‘4’) really reflect more agreement than ‘disagree’ (‘2’)? Or are agreeing and disagreeing with something not fundamentally different ideas? Semantically, different qualities can be easily merged into one conceptual dimension (e.g., semantic differentials; Snider and Osgood, 1969; Problem complexes §8 Naïve use of language-based methods and §9 Variable-based psychology and data-driven approaches). But what divisible properties could we identify in abstract, qualitatively heterogeneous (i.e., blended) concepts (Problem complex §7 Constructification)? When aggregating rating scores across items, could answering *1 x ‘strongly disagree’* (‘1’) *and 1 x ‘strongly agree’* (‘5’), thus, having a split opinion or inversed item interpretation, really indicate (roughly) the *same* level of agreement (averaging ‘3’) like answering *2 x ‘neither disagree nor agree’* (‘3’), thus having ‘no opinion’? The logico-semantic meanings of verbal answer categories—even if just ordinally conceived—are clearly discordant with the quantitative meanings that are commonly ascribed to the numerical scores into which they are recoded (Uher, 2022). Indeed, raters’ reasons for ticking off answer boxes are often not quantitative at all but rather trivial and thus different from researchers’ ‘scale’ interpretations (Problem complex §1a Researchers’ own role in their research; Uher, 2018a).

The ease of applying Steven’s ‘scale’ categories to rating ‘scales’ led psychologists to overlook that just (1) specifying a *structural data format* (e.g., five values) and (2) assigning to these values a particular *conceptual data format* (e.g., ordinality) neither enables the (3) necessary traceable *empirical interaction* with the measurand (data generation traceability) nor does it (4) specify a *conventionally agreed reference quantity* determining the assigned values’ quantitative meaning (numerical traceability). Measurement scales,^36^ by contrast, must fulfil all these four methodological functions, which are needed at different stages of a measurement process and therefore not interchangeable (for details and the disparate meanings and uses of ‘scales’ and ‘units’; see Uher, 2022).

These practices entail that, with rating ‘scales’, causal measurand–result connections (data generation traceability) cannot be established, precluding the results’ attribution to the measurands (e.g., the researchees). Without traceable connections to known quantities (numerical traceability), rating scores have no publicly interpretable quantitative meaning either. The only option to create meaning for such scores is their between-case comparison. This is why statistics became essential to implement quantitative approaches in psychology.

### Problem complex §11. Statisticism: Result-based data generation, methodomorphism and pragmatic quantification instead of measurement

Statistical methods enabling between-case analyses quickly became increasingly complex and a discipline of its own. Indeed, for many psychologists, statistical analyses seem to have become more of an end in itself than a means for analysing data obtained to explore questions and to solve problems (Brower, 1949; Lamiell, 2019). This has led to

… the syndrome that I have come to call *statisticism*: the notion that computing is synonymous with doing research, the naïve faith that statistics is a complete or sufficient basis for scientific methodology, the superstition that statistical formulas exist for evaluating such things as the relative merits of different substantive theories or the ‘importance’ of the causes of a ‘dependent variable’; and the delusion that decomposing the covariations of some arbitrary and haphazardly assembled collection of variables can somehow justify not only a ‘causal model’ but also, praise a mark, a measurement model’. (Duncan, 1984, p. 226; italics added; similarly, Lamiell, 2003, 2013).

Statistical methods build on particular theoretical assumptions. Their contributions to the results cannot be separated from those of the phenomena analysed. Statistical theories may therefore impose structures onto the data that, if erroneously attributed to the study phenomena, may influence and limit the concepts and theories that psychologists develop about these phenomena (Axiom 3). This *methodomorphism* (Danziger, 1985a) is a further instance where study phenomena are substituted with knowledge unrelated to them (Problem complex §1d Psychologists’ own role in their research), thereby conflating study phenomena with study means—psychologists’ cardinal error.

**Figure 9:**
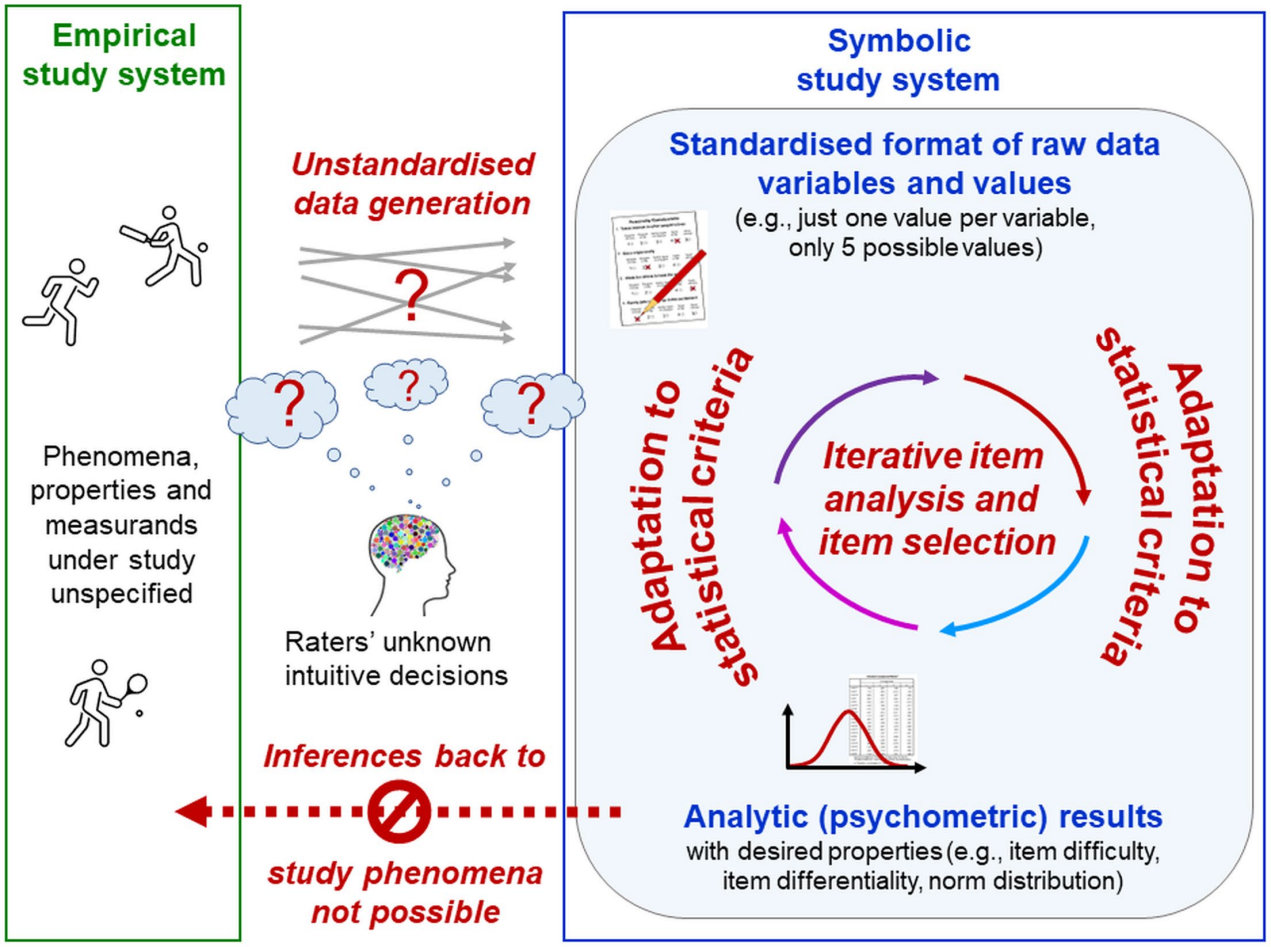
Result-based data generation enabling only pragmatic quantification. Psychometric ‘scale’ development involves iterative processes of item analysis and item selection, in which only those items are retained that allow the generation of data that differentiate well (e.g., item discrimination) and consistently between cases (reliability) and in ways considered meaningful (validity). This *result-dependent data generation*, however, aligns both the data generation and the analytical results to statistical criteria rather than to the study phenomena’s properties. This may be useful for pragmatic purposes but it precludes inferences from the analytical results obtained back to the phenomena, properties and measurands under study. These inferences are additionally compromised by the unknown intuitive decisions that raters are making when they generate the data before these are being psychometrically analysed and modelled.

Specifically, psychometricians develop rating ‘scales’ (amongst others) enabling the generation of numerical scores that differentiate well (e.g., item discrimination) and consistently between cases (reliability) and in ways considered meaningful (validity). This *result-dependent data generation*, however, aligns both data generation and analytical results to statistical criteria rather than to the study phenomena’s properties (Figure 9; Uher et al., 2013a; Uher, 2021d). Common psychometric ‘quality’ criteria, such as inter-rater and internal reliabilities, concern relations between the generated scores but neither these scores’ relations to the measurands (data generation traceability) nor to known reference quantities defining their meaning (numerical traceability). Thus, to create quantitative meaning for rating scores through sample-level statistics, psychologists analyse the relations of scores obtained for *different* individuals, and thus *different* measurands. This basically means comparing scores with unknown quantity information in order to create quantitative meaning for them—a truly Münchhausenian^37^ effort—which therefore fails when all cases score the same. In measurement, by contrast, the measurand’s unknown quantity (e.g., an individual’s body weight) is compared with that of a known reference quantity (e.g., standard kilogram unit), which establishes the result’s conventionally agreed quantitative meaning (e.g., *how* heavy that is; numerical traceability; see Figure 8).

Internal reliability and the validity of rating ‘scales’ additionally concern relations of scores obtained for *different* items and *different* constructs describing *different* phenomena with *different* properties. Hence, psychological validity theories are about empirical relations of the study phenomena (e.g., those described in an ‘extraversion’ ‘scale’) with some *other* phenomena that are considered to be meaningfully related for some reason or purpose (e.g., job performance or phenomena described in another ‘extraversion’ ‘scale’). That is, psychological validity concepts are about relations between phenomena *of different qualities*, whereas measurement is about capturing *quantitative* (divisible) properties of *one specific* quality (e.g., mass; Problem complex §10 Quantificationism). Psychometric scores are useful to discriminate between responses in ways considered to be meaningful but at the expense of unknown relations to the actual measurands in the phenomena studied and of their unknown quantitative meaning. This *utility perspective* is inherent to rating ‘scale’ development and validation, and appropriate for pragmatic purposes (Dawes et al., 1989; Barrett, 2003). But this *pragmatic quantification* is not measurement and therefore neither justifies the results’ attribution to the measurands (e.g., individuals) nor does it establish their quantitative meaning (e.g., *how much* of *what* specifically is it?; Uher, 2021c,d, 2022).

The complex statistics used in quantitative psychology may obscure these problems because every statistical operation removes the analysed data further from the phenomena that they are meant to represent. The more complex the statistics, the more difficult it is to keep track of their connections–which, for rating data, are already seriously compromised (Problem complexes §1–§10)–and thus to check the appropriateness of analyses and interpretations regarding the actual study phenomena (Brower, 1949). Specifically, statistical scores (e.g., effect sizes, correlations) are abstract concepts that describe distributions patterns in a sample and that can therefore inform neither about each measurand’s quantity (e.g., single individuals’ body weight) nor about the meaning of the quantity determined for a measurand (e.g., *how heavy* that is). Statistics neither is measurement (Fisher, 2009) nor is it therefore needed; indeed, measurement has been successful long before statistics has been developed (Abran et al., 2012).

Psychologists’ focus on sample-level statistics also influenced their understanding of nomothetic approaches, which are needed to generalise knowledge about individuals.

### Problem complex §12. Nomotheticism: Sociological/ergodic fallacy and primacy of sample-based over case–by–case based nomothetic approaches

Individual differences (differential) research is a field of its own, introduced as the population-level investigation of individuals (*differential psychology*)—together with the field devoted to individual-level investigations (*personality psychology*)—by William Stern who founded both sub-disciplines and laid their methodological foundations (e.g., variable-oriented and individual-oriented approaches,^38^ Stern, 1900, 1911; see Lamiell, 2003, 2013). Stern recognised that *inter*-individual variation and *intra*-individual variation are equally important characteristics of psychical phenomena. But the American assessment industry, group-based experiments (Danziger, 1985b) and the necessity to pragmatically create quantitative meaning for otherwise meaningless rating scores (Uher, 2021d, 2022) entailed that differential approaches prevail in psychology’s terminology, concepts, practices and scientific standards since the early 20th century (Cronbach, 1957; Richters, 2021).

This shifted psychologists’ focus away from analysing psychical processes, necessarily located in the single individual, to analysing distribution patterns in populations (e.g., socio-demographically defined). Results were now presented as aggregate data obtained from many individuals (e.g., group averages) without analysing individual patterns (Danziger, 1985a). But instead of attributing their results to the samples analysed, psychologists continued to interpret them with regard to single individuals, which remained their (unlike sociologists’) focus of interest and theoretical unit of analysis. This entails the *sociological fallacy*, which arises from the failed consideration of individual-level characteristics when drawing inferences regarding the causes of group-level variability (Diez Roux, 2002).

This inferential fallacy required axiomatic acceptance of *ergodicity*, a property of stochastic processes and dynamic systems, which presumes isomorphisms between *inter*-individual (synchronic) and *intra*-individual (diachronic) variations. Ergodicity fits all invariant phenomena, which do not undergo change or development, and in which simultaneity and successivity are therefore equal. But ergodicity does not apply to psychologists’ study phenomena (Axiom 1) as has been proven by applying classical mathematical-statistical (ergodic) theorems—first derived in ergodic theory, a branch of mathematics originating in statistical physics, in the 1930s (Molenaar, 2008; Molenaar and Campbell, 2009; Valsiner, 2014). Why did this elude ‘quantitative’ psychologists, despite their keen interests in implementing mathematical approaches analogous to the physical sciences?

Psychologists’ common practice to deduce information about *intra*-individual processes from analyses of *inter*-individual variability builds on the *ergodic fallacy* (van Geert, 2011; Speelman and McGann, 2020). Its institutionalisation is most prominently reflected in the common conflation of personality psychology with differential psychology (Table 1), especially in American psychology, where individual differences are regularly equated with individuality (Lamiell, 2013; Uher, 2018c) and where differential analyses of rating data (using variable-oriented approaches) are used by default to study individual functioning and development. From ergodicity it follows, however, that findings from group comparisons or correlations can be generalised only if (1) each individual obeys the same statistical model (assumption of homogeneity) and if (2) the statistical properties (e.g., factor loadings) are the same at all points in time (assumption of stationarity). But, in psychology, these conditions are rarely met (Molenaar, 2004; Molenaar and Campbell, 2009; Salvatore and Valsiner, 2010).

**Figure 10:**
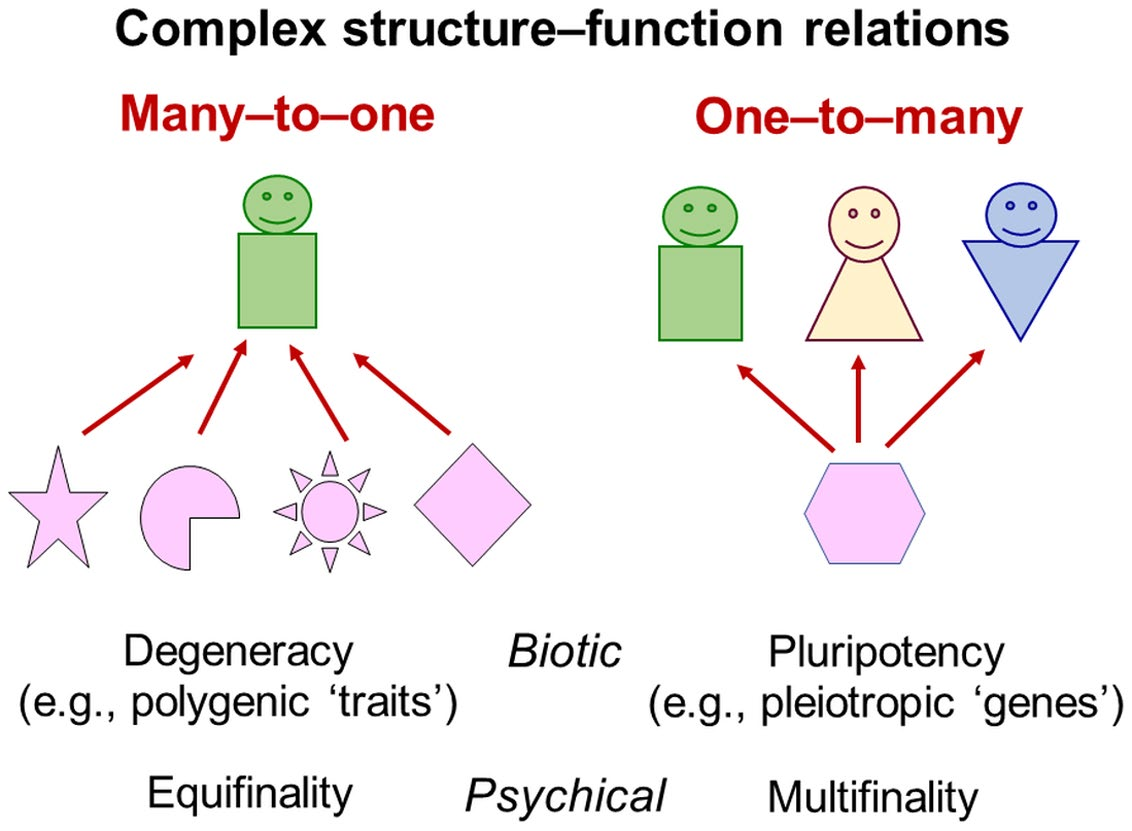
Fundamental design principles of complex living systems. Complex living systems feature both many–to–one structure–function relations (degeneracy, e.g., polygenic ‘traits’) and one–to–many structure–function relations (pluripotency, e.g., pleiotropic ‘genes’). This unifying explanatory principle also underlies the psychological concepts of the equifinality and multifinality of psychical phenomena.

The assumption of *psychical homogeneity*—that all individuals are the same—is logically necessary for sample–to–individual inferences and pragmatically and methodically convenient (e.g., assuming raters’ standardised item interpretation). But it is invalidated already by ordinary everyday experience, not to mention a solid body of empirical and theoretical research (Richters, 2021). Indeed, *psychical heterogeneity* is the core idea of ‘personality’ constructs! The assumption of psychical homogeneity also contradicts *degeneracy*, the capacity of different (non-isomorphic) structural elements to contribute to or perform the same function—a fundamental design principle underlying all complex biological systems (Axiom 1). Degenerate systems feature both *many*–*to*–*one structure–function relations* (degeneracy, e.g., polygenic ‘traits’) and *one*–*to*–*many relations* (pluripotency, e.g., pleiotropic ‘genes’; Mason, 2010, 2015). This unifying explanatory principle underlies the psychological concepts of *equifinality* and *multifinality*—individuals’ capacities to leverage *different* psychical processes and structures to accomplish the *same* behavioural outcome, and vice versa, respectively (Figure 10; Richters, 2021).

**Figure 11:**
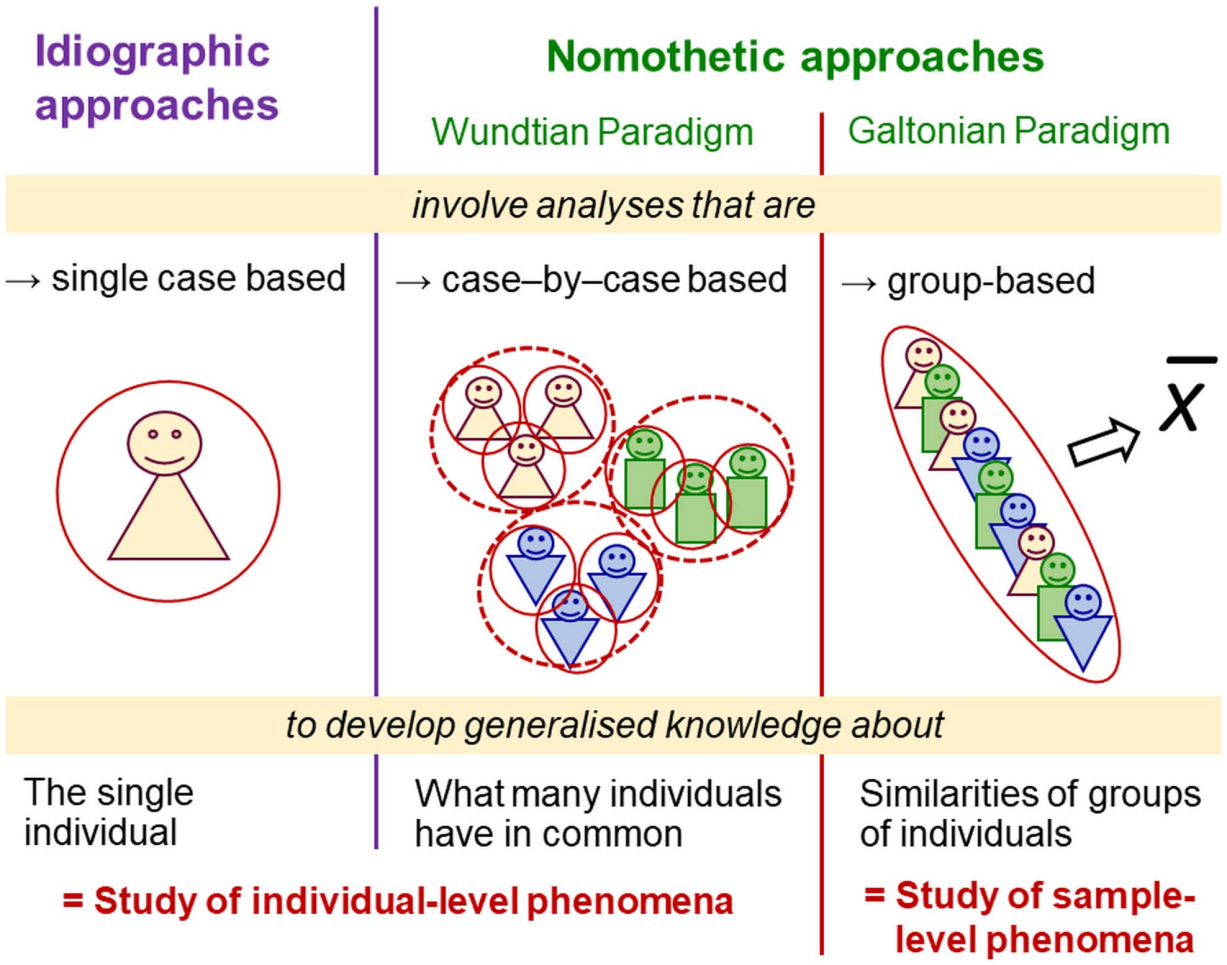
Strategies of knowledge generation. Idiographic approaches involve *single case based* analyses. In *case–by–case based nomothetic approaches* (Wundtian Paradigm), individuals are grouped by the commonalities and differences that they exhibit in the study phenomena, which enables the development of generalised knowledge and theories about intra-individual processes and functioning. In *group-based nomothetic approaches* (Galtonian Paradigm), by contrast, individuals are grouped by properties that the researchers find *a-priori* relevant, which inevitably influences and limits the concepts and theories that psychologists can develop about the phenomena studied (methodomorphism) and precludes identifying what individuals have indeed in common.

This highlights serious problems in the rationales commonly used to generalise findings in psychology. Specifically, the ergodic fallacy misleads many psychologists to understand nomothetic approaches (from Greek *nomos* for law) only in terms of *neo-Galtonian differential approaches* in which sample-level averages are studied and generalised to the individuals thus-summarised (Figure 11; Danziger, 1985b, 1990). But aggregates are statistical constructions of numerical data, constellations of data points featuring structural patterns that have no inherent meaning or theoretical significance in themselves (Richters, 2021). Limiting research to *group-level analyses* intrinsically disconnects theory development from descriptions of real individuals and cannot reveal what is common to all. Sample-based nomothetic approaches have turned psychology into a science largely exploring populations rather than individuals—*psycho-demography* (Lamiell, 2018; Smedslund, 2021). They lead to findings and theories that are uninformative about individuals’ functioning and development (Danziger, 1990; Lamiell, 2003; Robinson, 2011; Vautier et al., 2014; Smedslund, 2016), an important point increasingly considered also in applied fields, such as decision-making research (Chen et al., 2020).

The often mis-conceived and partly polarised debates about *idiographic* versus *nomothetic strategies of knowledge generation* (Figure 11; Lamiell, 1998; Windelband, 1904/1998), driven by the imperative of establishing psychology as a nomothetic science, mislead many psychologists to overlook that, nomothetic approaches are ultimately based on idiographic approaches—because every science builds on single cases. *Idiographic approaches* (from Greek *ideos* for the peculiar) model local phenomena of single cases in their dynamic contexts. These can then be explored using *case*–*by*–*case based nomothetic analyses* to identify generalities that are, indeed, *common to all* cases (Figure 11). Thus, individuals are grouped on the basis of the commonalities and differences that they are shown to exhibit in the study phenomena and properties. Considering degeneracy (many–to–one and one–to–many structure–function relations), the thus-created groups of individuals can then, in turn, be further explored for underlying structures and processes and for commonalities and differences in them. This *Wundtian nomothetic approach*, because it is case–by–case based, allows researchers to develop generalised knowledge and theories about *intra*-individual processes and functioning (Lamiell, 2003; Salvatore and Valsiner, 2010; Robinson, 2011).

*Galtonian sample-based nomothetic approaches*, by contrast, involve analyses in which individuals are grouped by properties that the researchers find *a-priori* relevant (specified as ‘independent variables’, e.g., ‘sex/gender’, age), thereby again substituting their own (pre-analytical) knowledge for their study phenomena and assuming these could be understood in terms of categories readily available to the researchers (Problem complex §1d Psychologists’ own role in their research). This knowledge inevitably influences and limits the concepts and theories that psychologists develop about the phenomena studied—another instance of methodomorphism (Problem complex §11 Statisticism).

Rating data, given their inherently differential meaning and obscured relations to the study phenomena, contributed to the primacy of sample-based nomothetic approaches in psychology. To explore intra-individual processes, data generation methods able to capture intra-individual variability are needed but still not very common in psychology (Van Geert and Van Dijk, 2002; De Ruiter et al., 2017). With individual-/ person-oriented methods, by contrast, a solid portfolio of analytical methods, grounded in William Stern’s methodologies, has already been developed for implementing case–by–case based nomothetic approaches in empirical investigations (e.g., Bergman and Trost, 2006; von Eye and Bogat, 2006).

## Conclusions

Measurement is valued in science and society because it provides reliable, accurate and precise information. Measurement in physics is fairly complicated. But with ratings, so it seemed, psychologists have devised a method allowing them to ‘measure’, with ease and efficiency, almost anything describable in brief colloquial statements through laypeople’s judgements. Of course, physicists and psychologists study vastly different objects of research and these necessarily require different methods of research. Certainly, psychology does not need the level of measurement accuracy and precision required for sciences like physics, chemistry and medicine, where errors can lead to airplane crashes, explosions or drug overdoses. And yet, psychologists themselves draw explicit analogies to physical measurement (e.g., in conjoint or Rasch ‘measurement’; Trendler, 2019, 2022; Uher, 2021c) and they regard rating ‘scales’ as ‘measuring instruments’ with psycho-‘metric’ ‘precision’ (Simms et al., 2019) and able to determine judgement ‘accuracy’ (Kenny, 1991; Funder, 1995).

But does it matter if we call it ‘measurement’? It matters because, measurement is not just any activity to produce numerical data. Across all sciences and in society, measurement is regarded a structured documented process that justifies the results’ attribution to the measurands and establishes their publicly interpretable quantitative meaning. These criteria justify the high public trust placed in measurement (Haggerty and Porter, 1997). Psychologists’ ‘measurement’ jargon therefore invites serious jingle fallacies (same term, different concepts) that can mislead researchers, decision makers and laypeople alike. For example, when presented as results of ‘measurement’, even minor differences are interpreted as meaningful and attributed to the individuals studied (e.g., ‘sex/gender’ differences; Hyde, 2005). This can have serious consequences, such as when psychometrically determined IQ scores expressed to two-decimal place precision are used in decisions on the death penalty for offenders (Barrett, 2018)—although psychometric scores are mere pragmatic quantifications (Uher, 2021c) that require adjustment to be meaningful (Young et al., 2007; Flynn, 2012). It will only be a matter of time that psychometric scores are challenged in court like forensic psychologists’ and psychiatrists’ diagnostic practices have been before (Faust, 2012; Barrett, 2018).

Changing the definition of a key scientific activity, such as by ‘softening’ or ‘widening the definition of measurement for psychology (e.g., Finkelstein, 2003; Mari, 2013), cannot establish its comparability across the sciences—but undermines it (Uher, 2020b). Psychologists’ ‘measurement’ jargon and complex statistics gave them a false sense of advancement and of having established a solid scientific framework for their empirical research. Therefore, critical reflection about the meaningfulness and interpretation of the numerical scores produced and of the quantitative analyses applied to them seemed to have become obsolete. Indeed, rating ‘scales’ are used in virtually identical ways for almost a century now (Thurstone, 1928; Likert, 1932) and differential approaches still prevail. Yet psychology’s continued crises about its findings, theories and research practices testify to fundamental problems still unsolved. Current initiatives to tackle these problems (e.g., open and ‘meta’ science; Malich and Rehmann-Sutter, 2022) are all targeted at improving data analysis and interpretation (e.g., response coding and transformation; construct operationalisation and validity; statistical tests; Flake and Fried, 2020; Hardwicke et al., 2022). But rating ‘scales’ remained largely unchallenged. They have become even psychology’s standard method of data generation (Baumeister et al., 2007) although its philosophical and theoretical foundations—needed to justify this powerful status—have not been elaborated.

The present rigorous analyses of the metatheoretical and methodological foundations on which rating ‘scales’—by their very conception, design and application—are built, revealed a dense network of 12 complexes of problematic conceptions, misconceived assumptions, logical errors and faulty jargon (Figure 3). Ambiguous terms with disparate meanings create logical gaps that researchers intuitively bridge through a conceptual back-and-forth switching between the concepts conflated, thereby establishing an internal coherence that makes this network difficult to be identified and recognised by those (unwittingly) relying on it. Through the widespread and uncritical application of rating ‘scales’, these 12 problem complexes have become institutionalised in a wide range of research practices and therefore cannot be remedied with little quick fixes that many may hope for.

This leaves but one conclusion: Unless ratings are removed from psychology’s portfolio of research methods, its recurrent crises (e.g., replication, confidence, validation and generalisability) cannot be tackled. Ratings may be useful for pragmatic purposes in applied fields, but they preclude measurement and—far more importantly—they preclude the scientific exploration of psychology’s study phenomena and thus its development as a science.

## Recommendations: Directions for solving psychology’s crises

As sobering as this account may be, it also opens up directions for development that are needed to solve psychology’s crises holistically by getting to the root of the problems rather than just scratching on the surface as many previous proposals. In this final section, specific theoretical concepts, methodologies and methods are derived from each of the 12 problem complexes, guided by the TPS-Paradigm’s basic axioms—Complexity, Complementarity and Anthropogenicity—on which the present analyses are based. The solutions proposed and their implications are necessarily overlapping and should therefore be considered holistically across the entire network, although, for a better overview and to avoid redundancies, Table 2 outlines for each problem complex only key implications.

**Table 2:** The 12 problem complexes, their implementation in rating ‘scales’ and derivation of directions for possible solutions.

**Metatheoretical and methodological problem complexes**	**Implementation in rating ‘scales’**	**Axioms, theoretical concepts, methodologies and methods required to tackle these problems**
**Problem complex §1. Psychologists’ own role in their research: Unintended influences**		
Perspectival disparity between researcher and researchee together with *psychologists’ non-independence of their objects of research* (being individuals themselves) entail a confusion of psychologists’ own standpoint with that of the psychical phenomena studied. Central *fallacious assumptions:* §1a Intersubjective confusion §1b Attribution of reflectiveness §1c Ignoring study phenomena’s relevance in researchee’s horizon of their lifeworld §1d Substituting psychical phenomena with knowledge unrelated to hese phenomena §1e Preference of scientific account over that the researchee’s §1f Misleading availability of ordinary words.	Raters are assumed to understand and use rating ‘scales’ similar to the researchers, to consciously reflect on and experience the phenomena as described by the researchers, thus not expressed in their own words and ignoring substantial variations on raters’ part. Item contents are chosen to fit researchers’ questions, theories and methods (e.g., for ‘scale’ development); thereby aligning rating ‘instruments’ to this scientific knowledge rather than to the described phenomena and their relevance for the raters. Everyday language invites all kinds of inferential and attributional biases (Problem complex §8 Naïve use of language-based methods).	**Anthropogenicity (Axiom 3):** Reflect on basic assumptions that may (unintentionally) influence research and that may be (implicitly) attributed to others, especially assumptions encoded in everyday language, which may therefore influence understanding and interpretations of both researchers and researchees. Study the meanings that researchees construct for themselves and as expressed in their own words (e.g., open-ended answers analysed using semantic computer algorithms), rather than using identical wordings and ignoring their inherently flexible and context-depending meanings (Problem complex §8 Naïve use of language-based methods). Ask researchees to reflect on and specify the phenomenon considered, the meaning it has for them in given contexts, and what specifically they considered in their judgements (e.g., using techniques of participant validation).
**Problem complex §2. Beliefs in researchers’ objectivity: Illusions of scholarly distance**		
By virtue of being researchers, psychologists often regard themselves as objectively distanced from the researchees and the study phenomena. But being individuals themselves, they are not independent of their objects of research; their own positioning in the world therefore precludes the possibility of taking a neutral observer standpoint.	Online questionnaires maximally distance researchers from researchees; any contact is just only indirect and virtual. But lack of contact hinders researchers from becoming aware of and exploring possible differences in perspective, presumptions, interpretations (Problem complex §1 Psychologists’ own role in their research), thus precluding implementation of any corrective means as is needed to develop concepts of ‘objectivity’ given the peculiarities of psychical phenomena.	**Anthropogenicity (Axiom 3):** Reflect on and consider the own positioning in the world regarding the study phenomena and the researchees (e.g., using techniques to document reflexivity as in interpretive methods). Conceptualise the social encounter, which every study on individuals constitutes, to explore how researchers and researchees and their roles may influence the results. Study and treat researchees as individual beings, not as anonymous sources of information. Get in direct contact and actually see the researchees in person (just as medical doctors and researchers do).
**Problem complex §3. Mistaken dualistic views: Individuals as closed system**		
Thought from an observer standpoint, researchers conceive individuals as opposed to and thus separate from the conditions in which these are being studied (e.g., situations, environments). In such *dualistic concepts*, external contexts are categorised by the researchers and ascribed researcher-determined meanings that are assumed to be identical for all researchees, ignoring that their relevance and meaning depend on the given individual.	Common belief that raters would react to item stimuli that have standardised meanings as determined by the researchers and as needed for quantification (Problem complex §10 Quantifiationism) but ignoring substantial and context-dependent variations in raters’ understanding and interpretation of the rating ‘scales’ and the phenomena to which they may refer (Problem complex §1 Psychologists’ own role in their research). Raters’ responses are flexibly attributed particular meanings, in line with researchers’ particular theories, thus involving several of the psychologist’s fallacies (Problem complex §1 Psychologists’ own role in their research).	**Complexity theories (Axiom 1):** Conceive individuals as complex open systems able to organise themselves in their systemic interdependences with their external contexts. Use *inclusive concepts*, in which relevant parts of an individuals’ surrounding and their meanings are identified and conceived in dependence of the researchee’s characteristics (e.g., their perception and interpretation of these contexts); such as using *Brunswik’s ecological validity concept* (not to be mistaken as similarity of experimental with everyday life situations) to describe the informativeness that elements of the researchees’ external contexts have for them. Apply *dialogic and dialectic concepts and theories* to explore individuals’ meaning making, functioning and development in their contextual embeddedness.
**Problem complex §4. Lack of definition and theoretical distinction of study phenomena: Conceptual conflations and intersubjective confusions**		
Basic psychological terms and concepts are poorly defined and ambiguous, whilst specific terms and concepts proliferate, causing numerous jingle–jangle fallacies. Lack of differentiation between psychical and behavioural phenomena entails inferential biases, conflation of description and explanation (Table 1), inadequate method choices, and thus the impossibility to explore these phenomena’s relations with one another and with other kinds of phenomena.	Rating ‘scales’ are worded in everyday language, which entails imprecision, high degrees of inferentiality and frequent conflation of description with explanation (Problem complex §8 Naïve use of language-based methods). Many inferential items require raters to judge entities that are generally imperceptible, imperceptible in others, or no longer perceptible, thus forcing raters to rely on memories, beliefs and ideas, thus their everyday constructs (Problem complex §7 Constructification). Which particular phenomena raters actually consider in a rating and thus encode in the data, or if they process the item contents just on the mere conceptual-semantic level remains unknown.	**Complementarity (Axiom 2), Complexity theories (Axiom 1):** Develop frames of reference, methodological approaches and methods that are appropriate for the study phenomena (e.g., accessibility-based conceptual distinction of kinds of phenomena and therefrom derived classes of suitable methods). Ensure that the study phenomena actually occur during data generation and record their temporal, situational and interpersonal contexts, profiting from technical advancements in tracking methods (e.g., reality mining, ambulatory [ecological momentary] monitoring; Problem complex §5 Reductionism). This is essential to explore the complex interplays between different kinds of phenomena and their emergent features in higher-order and more complex phenomena (e.g., actions, language, ‘extended mind‘), embedded in multi-level contexts.
**Problem complex §5. Reductionism: Category mistakes, atomistic fallacy and decontextualisation**		
Incorrect reductions are frequently made in psychology (often in conjunction with Problem complex §4 Lack of definition and theoretical distinction of study phenomena): *Ontological reductionism*: The claim that complex phenomena could be described in terms of more fundamental ones. *Epistemological reductionism*: The claim that higher-level phenomena could be explained by lower-level phenomena; this entails category mistakes. *Methodological reductionism*: The claim that complex systems could be understood by dissecting them into their supposedly isolable building blocks (*elementarism*). This *decontextualisation* entails the *atomistic fallacy* (from information obtained at lower levels, incorrect inferences are made at higher levels of organisation). It also entails that the interrelations and functioning of the isolated elements in their systemic contexts cannot be studied. As a result, description is used as and conflated with explanation (Table 1).	Rating data are often erroneously interpreted as reflecting ‘psycho-physical mechanisms’, often called ‘traits‘, which are assumed to be adaptive, inherited and universal across cultures. Concepts are broken down into brief, colloquially worded, often decontextualised statements, presented in mixed order, which precludes holistic, contextualised considerations and allows only for abbreviated reflection. Rating items radically dissect and decontextualise information about the study phenomena, thus involving the atomistic fallacy and precluding investigations of structural and processual patterns as they occur in the study phenomena. Instead, these chunks of verbal information are put together using statistical procedures, thus using knowledge unrelated to the study phenomena (Problem complexes §1 Psychologists’ own role in their research; §10 Quantificationism and §11 Statisticism) and also unrelated to the contexts in which they occur and in which raters may consider them (Problem complex §3 Mistaken dualistic views).	**Complexity theories (Axiom 1):** Conceive individuals as living organisms, especially emergence and inseparability of the study phenomena from their contexts (Problem complex §3 Mistaken dualistic views) as needed for investigations of causes and explanations (rather than just structural descriptions). Develop and use *contextualised approaches and methods* to study phenomena in their relevant contexts both within the organism and embedded into their larger external systems (e.g., abiotic, biotic, social, cultural), such as using tracking technology and methods of ambulatory [ecological momentary] monitoring (Problem complexes §3 Mistaken dualistic views and §4 Lack of definition and theoretical distinction of study phenomena). Apply *mereological concepts* to study whole–part relations (e.g., principle of emergence, catalysis, apperception). Develop theories of *processual change and development* and *dynamic causal relations* (e.g., using theories of complex dynamic systems and transdisciplinarity), not just analytic theories describing elemental structures isolated from the contexts in which they occur (Problem complex §7 Constructification).
**Problem complex §6. Operationalism: Logical errors and impeded theory development**		
A frequent idea in psychology is that a study phenomenon’s meaning could be established through the operations needed for its investigation, manipulation or elicitation. This *logical error* conflates the study phenomena with the means of their investigation—psychologists’ cardinal error. It entails further logical errors (e.g., *cause*–*effect conflation*, Table 1), toolbox thinking, proliferation of overlapping construct, and adapting research topics to the methods, rather than vice versa. Operationalism conflates disparate scientific activities (e.g., definition, hypothesis testing, data generation), thereby distorting scientific concepts and procedures. This impedes development of both theories about study phenomena and theories about methods.	Rating methods enable the flexible design of operational procedures, ad libitum and at low cost for any topic describable in small chunks of colloquial language (“*anything goes*” research attitude). This constitutes *a-theoretical instrumentalism* but enables the large-scale data generation needed to statistically analyse the outcomes of redundant operations for consistency (reliability) and empirical convergence that are considered to be meaningful for some reason or purpose (validity; Problem complex §11 Statisticism). Constructs are often defined through correlating item ratings (e.g., using factor analysis), thereby conflating scientific definition with empirical investigation. Belief rating ‘scales’ that are nominally associated with phenomena (e.g., ‘anxiety scales’) could be a valid method to study them (*toolbox thinking*).	**Anthropogenicity (Axiom 3):** Reflect on everyday constructs and lay definitions and their possible impact on the constructs and definitions developed and used in scientific psychology. Reflect on how researchers’ practical engagement with and collective appraisal of reality influences the knowledge they develop about that reality (e.g., learn from the philosophers, historians and sociologists of science). Explore the ways in which a field’s key concepts are theory-laden, socially embedded and historically contingent by explicating basic (hidden) assumptions and studying the concepts’ history (i.e., using literature older than just 5-10 years). Develop philosophical and theoretical definitions specifying the essences of study phenomena (e.g., using philosophy of science approaches). Clearly distinguish these definitions from the operational procedures needed to empirically investigate the phenomena defined (procedurism). Advance both, theories about the study phenomena and theories about methods (Problem complexes §4 Lack of definition and theoretical distinction of study phenomena, §10 Quantificationism and §11 Statisticism).
**Problem complex §7. Constructification: Studying constructs without also studying their intended referents**		
Constructs are *conceptual systems* referring to entities (referents) considered to be meaningfully related but that actually (can) never occur all at once. Thus, constructs are only conceptual entities; this has three important implications: (1) Researchers can empirically study only some of a construct’s referents chosen as *indicators*. (2) Construct referents can be all kinds of entities (e.g., abiotic, biotic, psychical, social, cultural). Despite differences in their forms of occurrence and accessibility thus requiring different research methods, heterogeneous referents can be conceptually integrated through abstraction into *blended constructs* (e.g., emphasising aspects considered relevant). (3) Given (1) and (2), constructs imply *more (surplus) meaning* than their concrete indicators, which therefore cannot be reflected in constructs as individuals can perceive them at a given moment. Psychologists’ confusions arise from frequent *construct*–*referent conflation* (Table 1) and because constructs can be used as means of exploration (scientific constructs) but can also be explored in themselves (e.g., everyday constructs)—but, in a study, a given construct logically cannot be both. The constructs’ construal on different levels of abstraction entails *nested conceptual structures* (symbolised with words) with complex meanings and referents. This may entail, especially in language-based research (Problem complex §8 Naïve use of language-based methods), that researchers study referents that are constructs in themselves rather than the actual concrete phenomena of interest to which these constructs refer and which therefore remain unstudied—the fallacy of *constructification*.	Inferential rating items describe not concrete phenomena but constructs that refer to (often heterogeneous kinds of) study phenomena in more general and abstract terms (Problem complex §4 Lack of definition and theoretical distinction of study phenomena). Their contents therefore can be judged also on the mere conceptual–semantic level (Problem complex §8 Naïve use of language-based methods). But even for judging (e.g., the intensity of) specific phenomena (e.g., specific behavioural acts), if considered, raters must make implicit comparisons over occasions, thus retrospective considerations, if not also over different individuals and different phenomena (e.g., other behaviours). Thus, ratings are inherently based on abstractions and generalisations. This entails the fallacy of constructification—researchers study only the everyday constructs that raters have developed *about* the actual phenomena of interest but not these phenomena (e.g., behavioural and psychical processes) in themselves, which therefore remain unstudied.	**Complexity theories (Axiom 1), Anthropogenicity (Axiom 3):** Metatheoretically and theoretically define the study phenomena (e.g., constructs, psyche, behaviours, language, actions), considering their processual, irreversible nature, momentariness, dynamicity, intra-individual variability, subjectivity, uniqueness, equifinality and multifinality and contextual embeddedness (Problem complexes §3 Mistaken dualistic views, §4 Lack of definition and theoretical distinction of study phenomena and §5 Reductionism). Develop methodologies, methods of data generation and methods of data analyses that are suitable for studying these phenomena and these particular properties (e.g., accessibility, forms and contexts of occurrence; Problem complexes §3 Mistaken dualistic views, §4 Lack of definition and theoretical distinction of study phenomena and §5 Reductionism). Develop concepts and theories of functional and causal relations between phenomena, thus of processual change and development (e.g., using complex dynamic systems and transdisciplinary theories), not just analytic and descriptive theories of elemental structures described on abstract levels (e.g., in everyday constructs), which preclude investigations of underlying processes (Problem complex §5 Reductionism).
**Problem complex §8. Naïve use of language-based methods: Reification and studying merely linguistic propositions**		
Language is often equated with verbal behaviour and psychical phenomena, overlooking its complexity (e.g., semantic networks, arbitrariness, variations, socio-cultural conventions), and its inherently representational and composite nature (signifier–referent–meaning relations). The role of semiotic systems for enabling conceptualisation and abstraction is hardly considered, therefore intricacies of language-based methods often overlooked. This entails fallacies, such that signifiers are equated with their meanings or their referents (psychologists’ cardinal error; Table 1) or linguistic abstractions are reified as real concrete entities (e.g., ‘traits’; fallacy of misplaced concreteness), which may mislead to take descriptions of the study phenomena for their explanation, resulting in explanatory circularity (Table 1). These fallacies also entail risks for studying merely linguistic propositions rather than the designated phenomena in themselves (Problem complex §7 Constructification).	Rating items are often thought to reflect standardised meanings (signifier–meaning conflation) or are equated with the phenomena they describe (signifier–referent conflation; Table 1). Variations in raters’ (and researchers’) item interpretation and use are often ignored although this entails that raters may consider in their ratings different meanings, thus also different phenomena than intended by researchers (Problem complex §1 Psychologists’ own role in their research). Researchers often conceive item responses as verbal behaviours, mixing up raters’ semantically guided meaning construction, everyday beliefs and hand movements for ticking off answer boxes (Problem complex §4 Lack of definition and theoretical distinction of study phenomena), leading to just pseudo-empirical findings. Rating-based research runs the risk of studying just linguistic propositions and the constructs designated (Problem complex §7 Constructification), both of which are often mistaken for the concrete phenomena to which they are intended to refer, thereby also often conflating description with explanation (Problem complexes §1 Psychologists’ own role in their research and §4 Lack of definition and theoretical distinction of study phenomena).	**Complexity theories (Axiom 1), Anthropogenicity (Axiom 3):** Develop concepts and theories that conceptualise human psychical life as intrinsically mediated by signs and language as inseparable from its users’ minds. This involves defining and differentiating language, psyche and behaviour to explore their interrelations, such as using complex dynamic system theories (Problem complex §4 Lack of definition and theoretical distinction of study phenomena) but also semiotic, semantic and pragmatic theories of language in exchange and collaboration with linguists. Develop and advance analytical approaches for language-based methods, which are inherently interpretive and cannot be standardised (e.g., using concepts of interpretive hermeneutics). Analyse textual materials produced in the researchee’s own words and ideally in everyday contexts (e.g., open-ended surveys, diaries, social media communications) so that researchers’ task is limited to selection and analysis of verbal materials (e.g., using semantic algorithms), without interfering in their production. Explore interpretations also on the individual level to avoid conducting mere semantic analyses and thus to study only codified conventional meaning structures (Problem complexes §11 Statisticism and §12 Nomotheticism).
**Problem complex §9. Variable-based psychology and data-driven approaches: Overlooking the semiotic nature of ‘data’**		
‘Data’ are interpreted as either the phenomena to be studied or the variables and values that are to be analysed in lieu of the actual study phenomena. Conflating these two meanings entails logical errors in analyses (Problem complex §6 Operationalism) and interpretation (psychologists’ cardinal error). The same conflation and logical errors occur for the term ‘variables’. Without sufficient consideration of data as sign (semiotic) systems (Problem complex §8 Naïve use of language-based methods), researchers overlook that data are inherently theory-laden (e.g., reflected in beliefs about ‘data-driven’ approaches). This entails that methodologies for data generation are hardly developed. Lack of transparency in data generation (representation theorems) can entail mismatches with methods of data analysis (e.g., uniqueness theorems) and result interpretations (Problem complex §10 Quantificationism). Analyses of collective variables (encoding blended constructs with heterogeneous referents) mislead statisticians’ result interpretations because it cannot be identified which referents and whether singly or collectively are relevant for a particular association found.	The dual function of rating ‘scales’ as description of the empirical study system and as symbolic (data) system promotes their conflation (psychologists’ cardinal error) and leaves the specification of each system and their relations (representation theorems) to raters’ implicit unknown decisions, thereby precluding transparency in data generation (and thus traceability; Problem complex §10 Quantificationism). Rating data are analysed only for the meanings that researchers consider (Problem complex §1 Psychologists’ own role in their research), ignoring their semantic fields of meaning (Problem complex §8 Naïve use of language-based methods), raters’ context-dependent meaning construction and particular interpretive perspective (Problem complex §3 Mistaken dualistic views), thus misinterpreting the information that particular rating data and the statistical results obtained from them can actually reflect.	**Complementarity (Axiom 2), Anthropogenicity (Axiom 3):** Develop a clear, unambiguous terminology, even if cumbersome, that avoids jingle–jangle and other fallacies and thus conflations. Develop methodologies of data generation (e.g., classes of methods determined by their modes of accessibility and forms of occurrence; Problem complexes §4 Lack of definition and theoretical distinction of study phenomena, §5 Reductionism and §7 Constructification) that are suited to establish transparency in the meaning of the data produced and their relations to their referents (e.g., data generation traceability, numerical traceability; Problem complex §10 Quantificationism). Carefully consider limitations in the interpretation of results from collective variables encoding (blended) constructs regarding their relations to their actual referents (Problem complex §7 Constructification).
**Problem complex §10. Quantificationism: Numeralisation instead of measurement**		
Quantification is widely believed to be essential for any science, a value in itself and generally better than qualitative findings—the belief of *quantificationism*. Still, elementary measurement concepts are largely unknown in psychology, such as *measurands*, *quantity* and *quality,* resulting in inadequate application of *quantity axioms.* Largely unknown are also concepts of *number* versus *numeral*, resulting in common *numeral*–*number conflation* (Table 1) and misguided interpretations of numerical data and their (quantitative) meanings. Operationalism entailed the erroneous belief that operations that yield convergent numerical results could provide evidence of quantitative properties in the study phenomena and could constitute valid ‘instruments’ for ‘measuring’ a construct (Problem complex §6 Operationalism). But traceable links from the measurand to both the result (*data generation traceability*) and a quantity of known defined meaning (*numerical traceability*) cannot be established, thus precluding the (1) results’ justified attribution to the measurands, and their (2) publicly interpretable quantitative meaning regarding the property studied—the two *criteria of measurement* (implicitly) applied across the sciences. Measurement scales must fulfil *four methodological functions,* which cannot be substituted for each other: they serve as (1) ‘instruments’ enabling empirical interactions with study phenomena and properties; (2) structural data format; (3) conceptual data format; and (4) conventionally agreed reference quantities.	Raters are required to assign a broad range of quantitative information flexibly to a fixed, narrow range of values; thus, to adapt their judgements to the ‘scale’ rather than to the study phenomena by constructing *different* quantitative meanings for the *same* answer category, which can distort and even inverse quantitative relations. Still, researchers rigidly score the verbal answer values in always the same ways, replacing words with numerals. This recoding ignores the logico-semantic meanings that the verbal categories could actually have as well as raters’ often rather trivial reasons for ticking off these categories, both of which are discordant with the quantitative meanings that researchers ascribe to the numerically recoded scores (Problem complex §1 Psychologists’ own role in their research). This recoding also takes off all information about the properties (‘x’ *of what*?) and quantities (*how much* of that is ‘x’) to which the scores may refer. This misleads researchers to assume these scores could be interpreted flexibly as indicating quantities of any property as required for their research (e.g., agreement as a property of the study phenomena in themselves rather than as part of the judgement process; Problem complex §1 Psychologists’ own role in their research). Steven’s ‘scale’ categories lead psychologists to overlook that just implementing structural and conceptual data formats is not measurement. Without traceable relations to the measurands and known quantity references, rating ‘scales’ enable just *numeralisation*—the creation of numerical scores that are neither attributable to the measurands (e.g., individuals) nor publicly interpretable (i.e., *how much/many of what*).	**Complementarity (Axiom 2), Anthropogenicity (Axiom 3):** Develop both theories of measurement and theories of the study phenomena (Problem complexes §4 Lack of definition and theoretical distinction of study phenomena, §5 Reductionism, §7 Constructification and §9 Variable-based psychology and data-driven approaches) that incorporate the philosophy-of-science foundations of quality and quantity in order (1) to theoretically justify the possibility or impossibility of identifying quantitative (divisible) properties in psychical and behavioural phenomena (their *measurability*) and of specifying for them known quantities that are suitable as reference standards; and (2) to elaborate these quantitative properties’ possible (ir)relevance to the given phenomena’s functioning and development and their interrelations with other phenomena. Derive from these two types of theories quantitative methodologies, methods (e.g., using fuzzy categories as in behavioural coding) and models (e.g., based on computerised algorithms) that are adapted to the study phenomena and their particular properties and that allow traceable relations to the measurands and known quantity references to be established in empirical studies that can therefore yield results that are both attributable to measurands and publicly interpretable. Advance also non-quantitative methods of both data generation and data analysis to enable contextualised research on complex dynamic study phenomena (Problem complex §4 Lack of definition and theoretical distinction of study phenomena) as well as approaches of systematic and transparent interpretation (Problem complex §8 Naïve use of language-based methods), meta-synthesis and meta-theorising, which are needed for concept development. Profit from the expertise of the social, health and other sciences and their enormous portfolio of pertinent methodologies and methods many of which are still largely unknown in psychology.
**Problem complex §11. Statisticism: Result-based data generation, methodomorphism and pragmatic quantification instead of measurement**		
Without traceable relations to measurands and to known quantities, quantitative meaning for scores (Problem complex §10 Quantificationism) can be created only through between-case comparisons, making sample-level statistics essential for implementing quantitative approaches in psychology. But this means comparing scores with unknown quantity information to create quantitative meaning for them, which fails if all scores are the same and may be useful for pragmatic purposes only. Statistics is often regarded an end it itself and mistaken for the basis of science (and measurement)—a notion called *statisticism*. Statistics are based on theories and assumptions, which impose structures onto the data that cannot be separated from those of the study phenomena and that may, if erroneously attributed onto these latter, bias pertinent concepts and theories—the fallacy of *methodomorphism.* The more complex statistical methods are, the more obscured become the statistical results’ relations to the actual study phenomena; this makes it difficult to scrutinise the adequacy of analytical tests and the appropriateness of interpretations. Sample-level statistics (e.g., effect sizes) are abstract parameters describing distribution patterns in a sample but they can neither be attributed to the samples’ single measurands (e.g., single individuals’ body height; Problem complex §12 Nomotheticism) nor create quantitative meaning for them (e.g., how tall is that?), thus cannot enable measurement.	To enable between-case comparisons, psychometricians develop rating ‘scales’ enabling the generation of scores that differentiate well (discrimination) and consistently (reliability) between cases and in ways considered meaningful (validity), such as by selecting items that produce norm-distributed values, show desired levels of item difficulty and item discrimination, or coherent score distributions across different items used for the same construct. But this adapts methods and results to statistical criteria and theories rather than to properties of the actual study phenomena (Problem complex §1d Psychologists’ own role in their research); thus enabling only *result-dependent data generation* but not measurement. Psychological validity concepts concern relations between phenomena of *different* qualities (e.g., those described in items used for different, theoretically (un-)related constructs or real-world outcomes like health or job performances; Problem complex §6 Operationalism). By contrast, measurement is about capturing *quantitative (divisible)* properties *of one specific defined quality.*	**Complexity theories (Axiom 1), Anthropogenicity (Axiom 3):** Use the methodological rationales underlying the principles of data generation traceability and numerical traceability, specifying the relations between empirical and symbolic study system (Problem complex §10 Quantificationism), to develop analogous principles of *data analysis traceability* specifying general rationales underlying the transformations that different kinds of analytical methods make within the symbolic study system (e.g., abstracting from uniqueness theorems and specific statistical theories). Such general principles will help establish transparency in the analytical results’ relations to the original raw data with regard to the information that these reflect about the measurands and their qualitative and quantitative meanings (e.g., rationales for grouping cases and choosing units of aggregation), thereby guiding researchers’ result interpretation with a clear focus on the empirical study system (Problem complex §12 Nomotheticism; Figures 6, 8). Use and further develop simpler statistical procedures that remove themselves only slightly from the original data, enabling meaningful interpretation regarding the samples (e.g., groups of individuals or repeated observations of single individuals) analysed (Problem complex §12 Nomotheticism). Linear analyses of sample-level convergence (e.g., factorial analysis), by contrast, depart very far from the original data and involve more assumptions that are not explicitly considered in the formal model and result interpretation and that cannot explore the nonlinear relations found in living organisms. Apply and advance knowledge of qualitative mathematics and other models needed to analyse dynamic processes.
**Problem complex §12. Nomotheticism: Sociological/ergodic fallacy and primacy of sample-based over case**–**by**–**case based nomothetic approaches**		
Many psychologists erroneously believe that structures of *inter*-individual differences could be informative about *intra*-individual functioning and development. Therefore, *sample-based nomothetic* (variable-oriented) approaches are widely used in which individuals are grouped by properties that the researchers find a-priori meaningful (Problem complexes §1 Psychologists’ own role in their research and §11 Statisticism) and the aggregates of the groups thus-created are generalised to single individuals. But such inferences build on the *sociological/ergodic fallacy* because, in phenomena that change and develop, diachronic and synchronic variations are not isomorphic. Valid sample–to–individual inferences would logically require the assumptions of psychical homogeneity and stationarity—but these contradict the empirical data bases as well as fundamental design principles of complex living systems: many–to–one (degeneracy, equifinality) and one–to–many structure–function relations (pluripotency, multifinality). In *case*–*by*–*case based nomothetic* (individual-oriented) approaches, by contrast, individuals are grouped by their commonalities and differences in the study phenomena—by what is indeed common to all cases. Degeneracy and pluripotency can be studied by exploring these groups further for communalities and differences in their underlying intra-individual structures and processes, thereby linking individuals with theory development.	The differential focus is inherent to rating ‘scales’ because it is needed to create quantitative meaning for rating scores (Problem complexes §10 Quantificationism and §11 Statisticism). Therefore, sample-based nomothetic approaches have become the default approach for analysing rating data, which contributed to their primacy in psychological research. Sample-level aggregates and their structures are commonly attributed to the single individuals and erroneously used to derive theories about individual-level phenomena (e.g., in Five Factor theory in ‘personality’ research, between-individual differences are conceptualised as an explanation of intra-individual phenomena), ignoring that this is based on the ergodic fallacy and conflating description with explanation (Problem complex §8 Naïve use of language-based methods; Table 1).	Advance and develop methods that are suited to explore *intra-individual* processes, change and development (Problem complex §11 Statisticism), both methods of data collection (e.g., momentary and situated recording of behaviours, physiological responses and experiential reports in ambulatory monitoring) as well as methods of data analysis (e.g., individual-oriented approaches like configurational frequency analysis; processual analyses) that allow researchers to adequately consider the non-ergodicity of psychical and behavioural phenomena. These should be integrated into suitable individual–socio–ecological frames of reference that need to be developed for the contextualised in-depth exploration of individuals using (instead of inductive differential generalisation from large samples) abductive generalisation to create meaningful findings that allow researchers to develop theories about individuals, their functioning and development.

For a better overview, relevant references are not included here but provided in the main text.

Finally, the author would like to offer some overarching general conclusions about key directions of development in psychology that go beyond the 12 problem complexes; detailed elaborations will be published elsewhere.^39^

### Make reflexivity a key qualification of any researcher

No research, including the author’s, is free of conceptual errors—simply because errors are human and science is inherently anthropogenic (Axiom 3). Recognising errors in one’s own thinking requires self-reflection about one’s own positioning as human being in the world and in one’s own research work (Bohr, 1937; Danziger, 1985b). Reflexivity should therefore be (re-)established as a basic skill of every researcher (Fahrenberg, 2013; Marsico et al., 2015). This requires reflection also about the embeddedness of science in its societal, political and historical contexts in general (Fleck, 1935; Kuhn, 1962) and in one’s own field of research in particular. When some psychology journals demanded citations to be limited to publications from just the last 5–10 years, the history of thought of key psychological concepts, theories and methods got partly out of sight. Knowing more about their origins and the contexts in which they had had once been created, will empower psychologists to critically reflect on the (implicit) foundations of their established research practices.

### Make explicit and elaborate the own metatheory and an unambiguous terminology

To establish psychology as a science, psychologists must take intellectual responsibility for its metatheoretical foundations (Vautier et al., 2014). Critical reflection and controversial debate presuppose that basic assumptions are made explicit (Axiom 3). This is a laborious task. It requires precise definitions, terminologies and concepts that cannot build on everyday language and that enable the crucial distinction between the study phenomena and the means of their exploration—thus, to avoid psychologists’ cardinal error. Such conceptual developments take far more efforts and time than empirical studies, on which currently most psychologists focus their research activities. But this conceptual research is necessary to identify inconsistencies and mismatches (see, e.g., Lilienfeld et al., 2015; Slaney and Garcia, 2015; Uher, 2021c,d), to trace theories and concepts to their origins, and to scrutinise their (meanwhile) often hidden underlying assumptions in order to enable reappraisal, critical reflection and even radical change and renewal (Danziger, 1990; Bennett and Hacker, 2003; Valsiner, 2012; Fahrenberg, 2013, 2015).

### Develop theories and concepts of the study phenomena

With rating ‘scales’, psychologists implemented a simplified but appealing image of natural science from which they created an equally simplified and appealing image of psychology as a science, but which cannot meet the complexities of its subject matter (Mazur and Watzlawik, 2016). We need theories that allow us to conceive individuals as living beings, as open self-organising systems featuring complementary phenomena (Axiom 2) and dynamic interrelations across their multi-layered systemic contexts (Axiom 1)—that is, theories not simply of elemental properties and structures but of processes, relations, dynamicity, subjectivity, emergence, catalysis and transformation (Fahrenberg, 2013, 2015; Cabell and Valsiner, 2014; Salvatore, 2015; Toomela, 2021; Valsiner, 2021). To explore continuous, dynamic, unprecedented and creative change and development, we need not simplistic dualistic but inclusive concepts (Table 2; Valsiner, 1997) as well as dialogic and dialectic theories (Veraksa et al., 2022).

### Develop methodology—The philosophy and theories of research methods

Psychology has become, in parts, a mere craft—focussed on the technicalities of data analyses (Maslow, 1946; Brower, 1949; Toomela and Valsiner, 2010). It is replete with theories of statistical analysis but devoid of theories of quantitative data generation (Uher, 2018a, 2021a). A quantitative methodology, specifying rationales and basic principles for linking the study phenomena with formal and quantitative models of investigation is still missing—the principles of data generation traceability and numerical traceability (Uher, 2020b, 2022) can be just a start (e.g., principles of data *analysis* traceability; Table 2).

Psychologists have still hardly considered the philosophical underpinnings of what quality and quantity actually are and how they are related (Hartmann, 1964; Rudolph, 2013; Uher, 2018a). Their philosophy-of-science definition highlights that quantification is useful for exploring only a minority of psychology’s study phenomena. Change and development, the key characteristics of most of the phenomena studied in psychology, are not just more of the same but involve *qualitatively different* structures and processes (Axioms 1)—therefore, quantification is of limited value (Valsiner, 2017b). Rather than sample-based quantitative analysis and meta-analysis for exploring group-averages, we need case–by–case based nomothetic approaches to explore individual functioning and development (Salvatore and Valsiner, 2010) as well as methods of qualitative synthesis and meta-synthesis, which are also essential for concept and theory development (Sim and Mengshoel, 2022).

Language is essential for science because results can be interpreted and communicated only in language (Axiom 3; Wittgenstein, 1922; Bohr, 1937). Language is also central for studying many psychical phenomena (Vygotsky, 1962; Valsiner, 2001; Salvatore et al., 2019). Language-based methods will always be important means of psychological investigation. Therefore, psychologists should acquire at least some basic knowledge of semiotics and semantics. Indeed, semantic computer algorithms, for example, are efficient methods to analyse open-ended verbal responses that can replace rating ‘scales’ in large-scale inquiries (Arnulf et al., 2021; Smedslund, 2021).

Replicability of psychological interventions and their effectiveness is important. But current approaches to validity and replicability are just pragmatic, providing evidence only of utility. In lack of traceability to the study phenomena, they provide no explanations or theories why these interventions are useful and how their effects come about (Uher, 2021c,d). Without understanding the phenomena, the actual causes of repeatable findings—which may be completely unrelated to the study phenomena—cannot be explored. *Explanatory psychology* exploring the microgenetic, ontogenetic and phylogenetic development of its study phenomena requires identification of abstract principles (Valsiner, 2021), not just endless repetitions of scores produced in ways that remain opaque and lay psychological. Instead of merely accumulating empirical findings, crucial experiments the results of which require major theoretical changes are particularly illuminating (Valsiner, 2014).

### Develop new research methods appropriately adapted to the study phenomena

Scientific discoveries of lasting and stimulating value have always led to new method developments. Psychologists cannot hope to progress by continuously re-applying old techniques to new, unprecedented problems (Brower, 1949). Methods of data generation must be developed that are appropriate to the study phenomena’s particular modes of accessibility to researchers and researchees and that consider possible complementary relations (Axiom 2; Table 2; Uher, 2019). Suitable methods are needed both to capture and to analyse psychical phenomena’s key features, in particular, their intra-individual variation, dynamicity, ephemerality, irreversibility, uniqueness, subjectivity, equifinality and multifinality (Sato et al., 2009; Valsiner, 2017b).

Psychologists’ present knowledge base of mathematics is limited and outdated. They need to widen their considerations to mathematical systems that are suitable to arrive at generalised knowledge about complex dynamic phenomena (e.g., quantum probability, topology, knot theory; Rudolph, 2013). To enable measurement, processes must be devised that establish traceable relations both to the phenomena and properties under study and to their quantitative and qualitative meanings. This involves also elaborating—and respecting—inherent limitations in the measurability of many psychological study phenomena (Uher, 2020b, 2022).

### Learn from other disciplines and their advancements

The complex phenomena of psyche, behaviour and society play central roles in all domains of individuals’ lives. Psychology’s focus on individuals and these phenomena puts the discipline at the intersection of many other sciences and of philosophy (Uher, 2021b). Therefore, psychologists are uniquely positioned to collaborate with other disciplines and to learn from the philosophical perspectives, metatheories, methodologies and methods that they have developed from their particular perspectives and for their particular research questions. Transdisciplinary research plays an important role in these endeavours because it aims to develop unitary frameworks that transcend disciplinary boundaries (e.g., complexity theories, dialectic theories). It can therefore highlight connections, differences and communalities across sciences—and thus, promising starting points for cross-scientific exchange and collaboration.

“The list of mistakes presented here was not intended to be exhaustive, nor the proposed solutions to encompass all possibilities. The present aim was, merely, to alert colleagues about the existence of these fallacies, and to provide them with a source and a reference. Hopefully this work will help prevent perpetuating these … mistakes on the grounds that ‘this is how things have always been done’ and ‘no-one ever said it was wrong.’ Now, you know” (Vanrullen, 2011, p. 6).

## Data availability statement

The original contributions presented in this research are included in the article/Supplementary material, further inquiries can be directed to the corresponding author.

## Author contributions

The author confirms being the sole contributor of this work and has approved it for publication.

## Funding

This research was funded by a Marie Curie Fellowship of the European Commission’s FP7 Programme awarded to me (EC grant agreement number 629430).

## Conflict of Interest

The author declares that the research was conducted in the absence of any commercial or financial relationships that could be construed as a potential conflict of interest.

## Publisher’s note

All claims expressed in this article are solely those of the authors and do not necessarily represent those of their affiliated organizations, or those of the publisher, the editors and the reviewers. Any product that may be evaluated in this article, or claim that may be made by its manufacturer, is not guaranteed or endorsed by the publisher.

## Footnotes

^1^The study of situated and physically described behaviour as done, for example, in human ethology, child research and behavioural ecology. Not to be confused with behavioural economics, sometimes labelled ‘behavioural’ science, which largely relies on rating ‘scales’ and surveys.

^2^Metrology, the science of measurement, foundational for the physical sciences and engineering.

^3^The TPS-Paradigm has already been applied to integrate, expand and complement established (1) concepts of psyche, behaviour, language and contexts (e.g., Uher, 2013, 2016a,b); (2) concepts and methodologies for taxonomising and comparing individual differences in various kinds of phenomena within and across populations (e.g., Uher et al., 2013a; Uher, 2015b,c,d,e, 2018b), as well as (3) concepts and theories of data generation, quantification and measurement across the sciences (e.g., Uher, 2019, 2020a,b) and in quantitative psychology and psychometrics (e.g., Uher et al., 2013b; Uher and Visalberghi, 2016; Uher, 2018a, 2021c,d; see Footnote 39). http://researchonindividuals.org

^4^Different terms can denote the same concept (jangle fallacies; Kelley, 1927) and the same term can denote different concepts (jingle fallacies; Thorndike, 1903).

^5^The term psyche is conceived more broadly than mind, thus comprising non-mental phenomena as well.

^6^Analogously, we get vir*al* (but not viro*logical*) infections but we do vir*ological* research.

^7^In lexical approaches, the person-descriptive words in a language’s lexicon are used to categorise the individual differences considered to be most important in a sociolinguistic community (Allport and Odbert, 1936; Uher, 2015d).

^8^von Uexküll (1909) differentiated Umwelt (external context) as opposed to Aussenwelt (external surrounding).

^9^In the TPS-Paradigm, a *phenomenon* is defined as anything humans can perceive or (technically) make perceivable and/ or that humans can conceive of (Axiom 3; Uher, 2015c). This definition differs from previous definitions, such as Kant’s (1781/1998) phenomena–noumena distinction. Unlike the latter, it considers that appearances are never purely perceived as such but influenced by the perceivers’ (pre)conceptions. Any attempt for making such distinctions for epistemological purposes is affected by the same problem. What humans cannot conceive of, cannot be researched (Axiom 3; see Wittgenstein, 1922, on linguistic prerequisites). This is particularly important for research on individuals where many phenomena are studied directly without using technical instruments (Uher, 2019).

^10^Immediacy indicates absence of phenomena mediating their perception (Wundt, 1896).

^11^The TPS-Paradigm’s concepts have also been misunderstood at times as either behaviourist or ontologically dualist (e.g., by Franz, 2021), ignoring their philosophical and theoretical foundation in theories of complex dynamic systems and of complementary relations (Axioms 1, 2). The present improved description may hopefully clarify these misunderstandings.

^12^Constructs and concepts are both abstract ideas. Constructs tend to have more heterogeneous referents and therefore to be more abstract and complex. But attempts to clearly differentiate them are ultimately arbitrary.

^13^The term constructification is used because the term constructionism is already established for constructivist approaches in psychology and social sciences. Constructionism refers to an epistemology (e.g., Gergen, 1985); whereas constructification refers to the fallacy described above.

^14^With very few exceptions (e.g., iconicity, onomatopoeia, indexicality).

^15^See footnote 9.

^16^In semiotics, signifier-referent relations are explored in the field of *semantics*; signifier-meaning relations in *pragmatics*, and interrelations between the signifiers of different signs in *syntactics* (Morris, 1938; Rød, 2004).

^17^Semantic networks are a logic-based formalism to describe structures in the mental organisation of knowledge representations and information retrieval pathways (Pirnay-Dummer et al., 2012), whereby the mental association strength between concepts (studied, e.g., with reaction times) is illustrated in nodes and directed edges. These networks describe multidimensional associations between constructs and their nested conceptual structures—thus, the fields of meaning that are common in particular sociolinguistic communities.

^18^Reflected in some psychometricians’ statements, such as "what really matters in validity is how the test works, and this is […] a property […] of the measurement instrument itself (i.e., *of the concrete, physical thing that you can drop on your feet, rather than of a linguistic entity*)." (Borsboom et al., 2009, p. 149, italics added).

^19^Representational measurement theory, however, provides no concepts and procedures for implementing such theorems and does not consider important elements of measurement (e.g., error and accuracy) and is therefore, insufficient for measurement in itself (Borsboom and Scholten, 2008; Mari et al., 2017).

^20^By comparison, operations suited for observations of behaviours are far more constraint as they are bound to individuals’ bodies and the moments and situations in which they occur. Together with the strong fluctuations of behavioural phenomena, this requires intense observer training and makes observations costly and labour intense.

^21^These principles, elaborated in the TPS-Paradigm on the abstract methodological level, underlie the frameworks of physical measurement in metrology. Data generation traceability is not explicitly formulated in metrology but implied by the process structures established. Numerical traceability is the methodological principle underlying metrological traceability, which is foundational to the International System of Units (Système International d’Unités, SI). This system codifies the internationally established measurement units and their reference quantities together with conventional definitions of their interrelations (BIPM, 2006; Czichos, 2011; Uher, 2022).

^22^Previously called the set-theoretic and the algebraic requirements of measurement (e.g., in Uher, 2015c,d).

^23^Behavioural researchers (see footnote 1) know that behaviour cannot be measured in themselves—only specific behavioural acts shown by a specific individual at a particular time and place can be measured, for example, regarding their temporal (e.g., duration, frequency) or spatial properties (e.g., distance).

^24^With increasing knowledge, this also requires theoretical explanations of the interactions enabled by these operations, whereas initial stages of instrument development often rely on mere instrumentalist approaches.

^25^These are called calibration chains because, along the connections in the chain, they specify uncertainties as a quantitative indication of the measurement quality of a result to assess its reliability (JCGM100:2008, 2008).

^26^The history of measurement in physics and metrology shows that reference quantities can be defined arbitrarily, while still allowing the establishment of a coherent system through systematic unbroken connection networks. The knowledge gained from decreed measurement units and the advent of modern technologies ultimately allowed scientists to replace arbitrary definitions with artefact-free definitions that are based on natural constants and thus reproducible any time and place (e.g., meter by speed of light; BIPM, 2006; Quinn, 2010).

^27^Bounded value ranges are also used for some measurement units; but these are either repeating, and thus unlimited (e.g., clock time), or inherent to the target property (e.g., degrees in a circle). Other quantitative categories with bounded value ranges refer to specified samples of unlimited size (e.g., percentages, counted fractions), thus indicating quantity values that are traceable (Uher, 2022).

^28^Presenting raters with numerical rather than verbal answer categories does not solve the problems discussed here but only shifts the execution of this recoding to implicit, unknown considerations by raters.

^29^These are Arabic, Roman and Tamil numerals all indicating ‘four’. Tally marks (e.g., ‘IIII’) are also signifiers.

^30^Numeral–number conflation is also widespread in everyday life.

^31^Interestingly, Stevens’ definition of ‘measurement’ correctly refers to the assignment of numerals, not numbers (Stevens, 1946, p. 667).

^32^In metrology, *measurement accuracy* denotes how closely the determined value agrees with the measurand’s true quantity value (e.g., someone’s true body height); *precision* denotes closeness between values obtained in replicate measurements (e.g., body height determined using the same or different yardsticks). *Measurement error* is the difference between measured and true value; *uncertainty* describes the dispersion of values attributed to a measurand, thus the reliability by which the measured value can be attributed to the true value (JCGM200:2012, 2012).

^33^Johnson (1943) similarly highlighted that numerals [he wrote ‘numbers’] are often treated without regard to either their functions (e.g., nominal, ordinal, cardinal) or their characteristics of abstractness or concreteness, which he referred to as *numerology*.

^34^Therefore, so-called ‘quantitative’ and ‘qualitative’ methods or data cannot be complementary to each other (Axiom 2), as recently suggested (e.g., Ramlo, 2021).

^35^For an English translation, see Michell and Ernst (1996).

^36^For this reason, ‘scales’ that to not fulfil all four methodological functions are put in quotation marks in the TPS-Paradigm.

^37^Referring to the famous story of Baron Münchhausen who pulled himself and the horse on which he was sitting out of a swamp by his own hair.

^38^Data matrices of *X_i_* individuals by *Y_j_* variables can be explored from two orthogonal viewpoints. *Variable-oriented approaches* focus on variables and study the distributions of their values across all *i* individuals, thus, exploring populations and patterns of between-individual differences but not single individuals. *Individual-/person-oriented approaches*, by contrast, focus on individuals and study each individual’s values on all *j* variables, thus exploring individual configurations of values across different variables that can be illustrated as a profile, which reflects a property of the individual but not of the population (Bergman and Trost, 2006; Uher, 2011).

^39^Uher, J. (in preparation). *Overcoming Method-Centrism in Psychology – Beyond Rating Scales*. (Switzerland: Springer Nature).
